# Innovative Clinical Trial Approach for Evaluating Digital Medical Devices Under European Fast‐Track Regulatory Frameworks

**DOI:** 10.1002/sim.70572

**Published:** 2026-05-05

**Authors:** Moreno Ursino, Sandrine Boulet, Corinne Collignon, Florence Francis‐Oliviero, Edouard Lhomme, Raphaël Porcher, Florence Saillour, Gaël Varoquaux, Vincent Vercamer, Rodolphe Thiébaut, Sarah Zohar

**Affiliations:** ^1^ Inserm, Inria, Université Paris Cité UMRS 1346, HeKA Paris France; ^2^ Haute Autorité de santé Saint Denis France; ^3^ Université de Bordeaux ISPED, INSERM, Bordeaux Population Health Research Center, U1219 Bordeaux France; ^4^ Service d'Information Médicale CHU de Bordeaux Bordeaux France; ^5^ Inria SISTM Bordeaux France; ^6^ Université Paris Cité and Université Sorbonne Paris Nord Inserm, INRAE, Center for Research in Epidemiology and Statistics (CRESS) Paris France; ^7^ Inria Soda Palaiseau France; ^8^ Digital Health Delegation French Ministry of Health Paris France

**Keywords:** Bayesian, interim analysis, meta‐analysis, randomized clinical trial, real‐world data

## Abstract

To address patient demand for rapid access to innovative digital medical devices (DMDs), several health technology assessment (HTA) authorities in European Union countries provide transitional or provisional access and reimbursement pathways. These pathways are available when only incomplete clinical trial data are accessible, and significant uncertainty remains regarding the clinical benefits, even after CE (European conformity) marking has been obtained. Once manufacturers complete the clinical studies, additional real‐world data (RWD) may become available as a result of the device's use in the target population. Consequently, regulators can draw on both sources of information to support their final decision‐making processes. For a statistically principled evaluation of such settings, we propose a statistical framework suitable for DMD evaluation under European HTA fast‐track requirements, integrating both clinical trial data and RWD. The framework consists of three key steps: (1) an interim analysis of clinical trial data, which can support temporary regulatory authorization and enable the collection of RWD; (2) a final analysis of the clinical trial data; and (3) a meta‐analysis combining the clinical trial data and RWD, contingent upon obtaining temporary authorization. To optimize the timing of the interim analysis and the application for temporary authorization, we introduce several metrics. The proposed framework was assessed by means of an extensive simulation study. This framework should be complemented by a post‐market evaluation of the DMD once it has been widely adopted, aligning with the principles of phase IV studies.

**Abbreviations**
CE: European conformity
CP: conditional power
DMD: digital medical device
EU: European Union
FDA: U.S. Food and Drug Administration
HTA: health technology assessment
NNHM: normal‐normal hierarchical model
PECAN: *prise en charge anticipée numérique*
PP: predictive power
RCT: randomized clinical trial
RWD: real‐world data
SaMD: software as a medical device
SiMD: software in a medical device

## 1 Introduction

Digital medical devices (DMDs) have the potential to reshape health prevention and care. Defined under the European Union (EU) regulation 2017/745, DMDs can be designed to prevent, diagnose, monitor, treat, or alleviate the effects of diseases. DMDs can provide benefits to individual patients as well as the broader healthcare system. This class of device includes smartphone apps, standalone software, online tools and any algorithm using data from medical devices such as medical imaging devices, sensors or monitors. Examples of DMDs include clinical decision support systems used by healthcare professionals, physical devices paired with software, such as closed‐loop insulin pumps [1], and apps for monitoring chronic diseases.

The U.S. Food and Drug Administration (FDA) categorizes these health technologies into two types: Software as a Medical Device (SaMD), which is software intended for one or more medical purposes and operates without being part of a physical medical device, and Software in a Medical Device (SiMD), which is software embedded in a physical medical device, essential for its function and performance. SiMD cannot work on its own and depends on the associated device to fulfill its purpose. For the sake of simplicity, this article uses the broader term DMDs to include both SaMD and SiMD.

The rapid development of artificial intelligence algorithms has transformed the DMD landscape by (1) deriving new and important insights from the vast amount of data generated during the delivery of every day health care, (2) enabling the personalization of patients' treatment, (3) empowering patients by changing the way they are monitored and followed, and much more. They have the potential to improve the quality, safety and healthcare pathways [2, 3, 4, 5].

DMDs are classified based on a risk‐based system that takes the human body's vulnerability and the potential risks of each device into consideration [6]. For medium‐ and high‐risk DMDs, after obtaining the CE marking, clinical evaluation through clinical studies, either interventional or observational in real‐life settings, is mandatory prior to applying for a Health Technology Assessment (HTA). However, conducting such clinical studies takes time while the technology on which DMDs are based on, especially with regard to machine learning or deep learning algorithms, evolves rapidly. This highlights two contradictory priorities: the need for rigorous clinical evaluation to ensure the safety and efficacy of DMDs, and the legitimate expectation of users (such as patients or medical staff) of having rapid access to innovative solutions. Like other health products, as in the case of drug evaluation, medical devices are assessed through clinical trials focusing on patient‐oriented clinical endpoints. However, the design and execution of interventional trials are time‐intensive, often spanning from the initial planning phase to the final patient inclusion and data analysis, which can take more than a year or even several years in some cases. While randomized clinical trials (RCTs) are considered the gold standard, observational studies, although less expensive, are often subject to biases and are not accepted as the same level of conclusive proof.

### 1.1 Motivation–European HTA Fast‐Track Frameworks for DMDs Reimbursement

Growing expectations for rapid reimbursement decisions regarding DMDs are reshaping HTA processes: several HTA agencies across EU countries have integrated, or are in the process of integrating, rapid access procedures for health technologies [7]. While evidence‐based medicine remains the standard for reimbursement decisions, the introduction of conditional fast‐track pathways – associated with early access and reimbursement – offers an appealing solution for patients, healthcare professionals, and the medical device industry. For instance, the French fast‐track pathway, known as PECAN (*prise en charge anticipée numérique*, early access to reimbursement for DMDs) [8], features a one‐year provisional reimbursement period to provide rapid patient access to digital health solutions. DMDs are reimbursed by the national healthcare system for one year based on preliminary evidence of potential benefit. Preliminary analyses of available data from ongoing studies (which are not necessarily randomized) are evaluated by the HTA body, and results are expected before the final HTA assessment for permanent listing at the end of the one‐year period. As a result, this process encourages the submission of partial study results to the HTA body, acknowledging the inherent uncertainty this approach presents for decision makers. Despite this, authorities must have sufficient data to justify provisional reimbursement and user access, even though complete clinical evidence may not yet be available. Once this transitional year is completed, manufacturers should have finished the clinical study and may also have collected real‐world data (RWD) derived from the DMD's use in the population. Consequently, regulators could benefit from two sources of information for their final decision‐making, whereas previously their analyses were based solely on clinical trial data.

**FIGURE 1:**
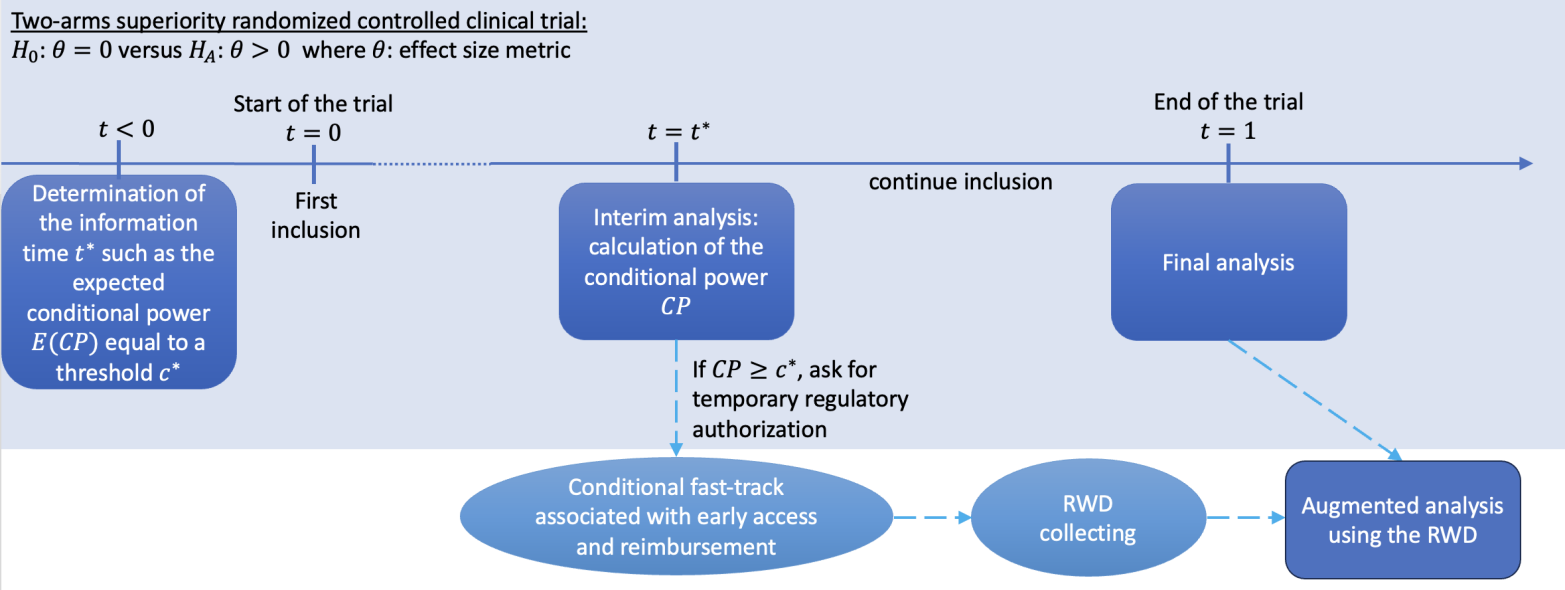
Schema of the proposed approach to facilitate more rigorous evaluation of DMDs under European fast‐track requirements. This approach is divided into three steps: (1) interim analysis conducted during the clinical trial, where results may support temporary regulatory authorization and enable real‐world data collection; (2) final analysis of the clinical trial; (3) meta‐analysis combining clinical trial data and real‐world data if temporary authorization has been granted. Conditional power is used here as an example, but other similar metrics, such as predictive power, can also be applied.

In this article, we propose a statistical framework for evaluating DMDs under these fast‐track requirements (see Figure 1). This framework is based on the context where an initial two‐arm RCT design for superiority is planned, including an interim analysis. Unlike traditional sequential analysis, this interim analysis will determine whether to seek regulatory temporary authorization rather than deciding on futility or continuation. Thus, type I error (considering the entire clinical analysis) is not affected by a multiplicity problem. If regulatory temporary authorization is pursued at the interim stage, additional prospective observational data (or RWD) on the same population could be available at the time of trial's conclusion. Therefore, in addition to the complete analysis of the clinical study reported at the end of the trial, the final step of the framework will involve using the additional observational data from temporary authorization in the general population through augmented analysis, accounting for heterogeneity within data sources. The framework thus caters for the different needs: the interim analysis brings together safety and rapid access, while the final analysis ensures rigorous efficacy evaluation.

This article is organized as follows. The methods are presented in Section 2. Section 3 describes the simulation settings used to assess our methodology and an illustration of two simulated data sets is provided in Section 4. Since it is only recently that users can access certain new interventions without the entire clinical trial procedure having been completed, RWD obtained in parallel with the clinical trial are, for the moment, particularly limited. As a result, the new framework is only evaluated via an extensive simulation study. The simulation results are given in Section 5. Our conclusions are provided in Section 6.

## 2 Methods

We propose a framework divided into three steps: (1) an interim analysis during the clinical trial, the results of which may support temporary regulatory authorization and enable the collection of RWD; (2) a final analysis of the clinical trial; (3) an augmented analysis incorporating RWD, if temporary authorization has been granted. As outlined in the previous section, the interim analysis will determine whether to seek temporary regulatory authorization. We propose several metrics for deciding the timing of the interim analysis. If temporary authorization is requested and granted, the DMD can be used beyond the trial population, enabling data collection on the same population and endpoint in an observational setting. At the conclusion of the trial, the trial data will undergo final analysis. If temporary authorization was granted, a meta‐analysis could be conducted, integrating the resulting external RWD with the trial, to further strengthen the trial results. A suitable adjusted analysis must then be performed for the RWD, using methods such as propensity scores and/or matching techniques [9, 10]. In addition, the clinical trial and the RWD may not be directly comparable due to differences in population recruited (variations in patient characteristics, treatment settings, or data collection processes) [11]. Generalizability, or transportability, can be employed to align the RWD population more closely with the trial population, weighting, stratifying, or matching individuals [12, 13]. In the following sections, we outline the proposed metrics and methods to be utilized within the framework.

### 2.1 Clinical Evaluation With an Interim Analysis

Consider a two‐arm superiority randomized controlled clinical trial, involving a new intervention with a DMD and a control group, comprising a total sample size of n participants, to test the null hypothesis $H_{0}:\theta =0$ versus $H_{A}:\theta >0$, where θ represents an effect size metric, such as the mean difference in the primary endpoint between the intervention and control groups. Given a sample of size $n_{t}=t\;n$ at the interim analysis, t∈]0,1[ being the “information time”, define $G_{t}$ as an estimator for θ with its corresponding variance denoted as $k^{2}/t$, where $k^{2}$ is the variance of the estimator in the final analysis ($G_{1}$). Here we purposely consider only very general properties of the estimator: the framework is applicable to many different estimators. Table 1 shows some examples of estimators and definitions for continuous and binary endpoints. Under these conditions, the test statistic

(1) $$Z_{t}=\frac{G_{t}}{k}\sqrt{t}$$

typically follows a normal distribution $Z_{t}∼𝒩(\sqrt{t}\theta /k,1)$, which simplifies to $Z_{t}∼𝒩(0,1)$ under $H_{0}$. For the sake of simplicity, we consider only a single interim analysis at time t, but the framework can be easily extended for multiple interim analyses. The operating characteristics (such as the type I error, α, and the power, 1−β) of this testing procedure are determined by the joint distribution of the vector $Z={\left(Z_{t},Z_{1}\right)}^{T}$ under both the null hypothesis $H_{0}$ and the alternative hypothesis $H_{A}$ on a pre‐specified value $\theta_{A}$. The conditional power (CP) is defined as the probability of achieving statistical significance at the prespecified level α at the end of the study, given the results obtained so far and assuming the future data will follow a certain distribution, typically related to the original assumptions of the study. Mathematically, it can be expressed as:

(2) $$\text{CP}=\Phi \left(\frac{\sqrt{t}\;z_{t}+\theta^{\prime }(1-t)/k-z_{1-\alpha }}{\sqrt{1-t}}\right),$$

where Φ is the cumulative distribution function of a standard normal variable, $\theta^{\prime }$ is the estimated value of θ for the post‐interim period, $z_{t}$ is the observed value of $Z_{t}$ and $z_{1-\alpha }$ is the 1−α quantile for the standard normal distribution. Several choices of $\theta^{\prime }$ can be used in Equation (2), such as those associated with the alternative hypothesis or the value $g_{t}$ of the estimator $G_{t}$ at the time t of the analysis [14, 15, 16, 17].

**TABLE 1:** Examples of $g_{t}$ and k for different endpoints in a two‐arm trial (intervention (I) versus control (C)).

Continuous endpoints
Population means $\mu_{I}$ and $\mu_{C}$.
Test: $H_{0}:\mu_{I}-\mu_{C}=0$ versus $H_{A}:\mu_{I}-\mu_{C}>0$, with power computed at $\Delta_{A}$.
At interim analysis with total sample size $n_{t}$:
$g_{t}=d_{t}={\bar{x}}_{I,t}-{\bar{x}}_{C,t}$, where ${\bar{x}}_{I,t}$ and ${\bar{x}}_{C,t}$ are the sample means at interim.
$Z_{t}=\frac{G_{t}}{s_{n_{t}}/\sqrt{n_{t}}}$, where $s_{n_{t}}$ is the estimate of the pooled standard deviation at interim analysis.
$k=s_{n}/\sqrt{n}$.

Binary endpoints
Population proportions $\pi_{I}$ and $\pi_{C}$.
Test: $H_{0}:\pi_{I}-\pi_{C}=0$ versus $H_{A}:\pi_{I}-\pi_{C}>0$, with power computed at $\Delta_{A}$.
At interim analysis with total sample size $n_{t}$:
$g_{t}=d_{t}=p_{I,t}-p_{C,t}$, where $p_{I,t}$ and $p_{C,t}$ are the estimated proportions at interim.
$Z_{t}=\frac{G_{t}}{s_{n_{t}}/\sqrt{n_{t}}}$, where $s_{n_{t}}$ is the estimate of the pooled standard deviation at interim analysis.
$s_{n_{t}}^{2}=\frac{1}{2}(p_{I,t}(1-p_{I,t})+p_{C,t}(1-p_{C,t}))$.
$k=s_{n}/\sqrt{n}$.

*Note:* The notations $d_{t}$ and $\Delta_{A}$ are respectively used instead of $g_{t}$ and $\theta_{A}$ for the special case for which a difference is used as the estimator $G_{t}$.

On the other hand, the predictive power (PP) is the probability of trial success at completion after integrating over the uncertainty in the effect size of the empirical estimate, considering both the observed data and prior information. It is also linked to a Bayesian metric [14] and provides a probability distribution over possible future outcomes of the trial, accounting for the observed data. It can be formally defined as:

(3) $$\text{PP}=\Phi \left(\frac{\sqrt{t}\;z_{t}-t\;z_{1-\alpha }}{\sqrt{t\;(1-t)}}\right).$$

Derivations of CP (Equation (2)) and PP (Equation (3)) are available in Lan et al. [14]. Small differences in notation between this reference and our article are explained in Section 1 in the Supporting Information.

The core concept here is to leverage one of the two metrics introduced (CP in Equation (2) and PP in Equation (3)) in order to make critical decisions during the interim analysis of a clinical trial. Given the need for robust data in regulatory submissions, we recommend conducting the interim analysis only after a significant proportion of participants have been enrolled. This approach marks a departure from typical practices where CP or PP is assessed early in the recruitment process [18]. In contrast, we propose scheduling the interim analysis for the latter half of the recruitment period (t≥0.5), and setting a higher threshold, between 60% and 80%. As a reminder, the interim analysis is solely intended to decide whether to apply for regulatory temporary authorization, with no plans to halt the trial. Therefore, the time t can be chosen to achieve the desired conditional power $c^{*}$, such as $c^{*}=0.7$ or 0.8. In this case, we can compute the expected conditional power, defined as the a priori expectation of the probability of achieving statistical significance at the end of the trial, by integrating the conditional power under the empirical trend, that is, using $\theta^{\prime }=g_{t}$, over the $Z_{t}$ distribution under the alternative hypothesis, which is:

(4) $$E[CP]=\int \Phi \left(\frac{z_{t}/\sqrt{t}-z_{1-\alpha }}{\sqrt{1-t}}\right)f(z_{t}|\theta_{A})\;dz_{t},$$

where f denotes the density of the normal distribution $𝒩(\sqrt{t}\theta_{A}/k,1)$ and $\theta_{A}$ represents the desired θ under the alternative hypothesis $H_{A}$. Equating Equation (4) to $c^{*}$ allows us to compute the sample size $n_{t}=tn$ for the interim analysis. The same reasoning can be applied to the predictive power, giving:

(5) $$E[PP]=\int \Phi \left(\frac{\sqrt{t}z_{t}-tz_{1-\alpha }}{\sqrt{t(1-t)}}\right)f(z_{t}|\theta_{A})\;dz_{t}.$$

Similarly, the expected predictive power is obtained by further averaging the predictive power over the posterior uncertainty in the true effect size, that is, by integrating over both the predictive Z‐distribution and the effect‐size distribution. However, we may also be interested in performing the interim analysis when the probability of having a CP greater than a threshold $c^{*}$ is equal to a pre‐specified probability γ. Therefore, we are looking for the time t at which this condition is met, that is:

(6) $$\int I\left[\Phi \left(\frac{z_{t}/\sqrt{t}-z_{1-\alpha }}{\sqrt{1-t}}\right)\geq c^{*}\right]f(z_{t}|\theta_{A})\;dz_{t}=\gamma ,$$

where I[(expression)] represents the indicator function that gives 1 if the expression holds and 0 otherwise. Similarly, for the predictive power we have the following expression:

(7) $$\int I\left[\Phi \left(\frac{\sqrt{t}z_{t}-tz_{1-\alpha }}{\sqrt{t(1-t)}}\right)\geq c^{*}\right]f(z_{t}|\theta_{A})\;dz_{t}=\gamma .$$

**FIGURE 2:**
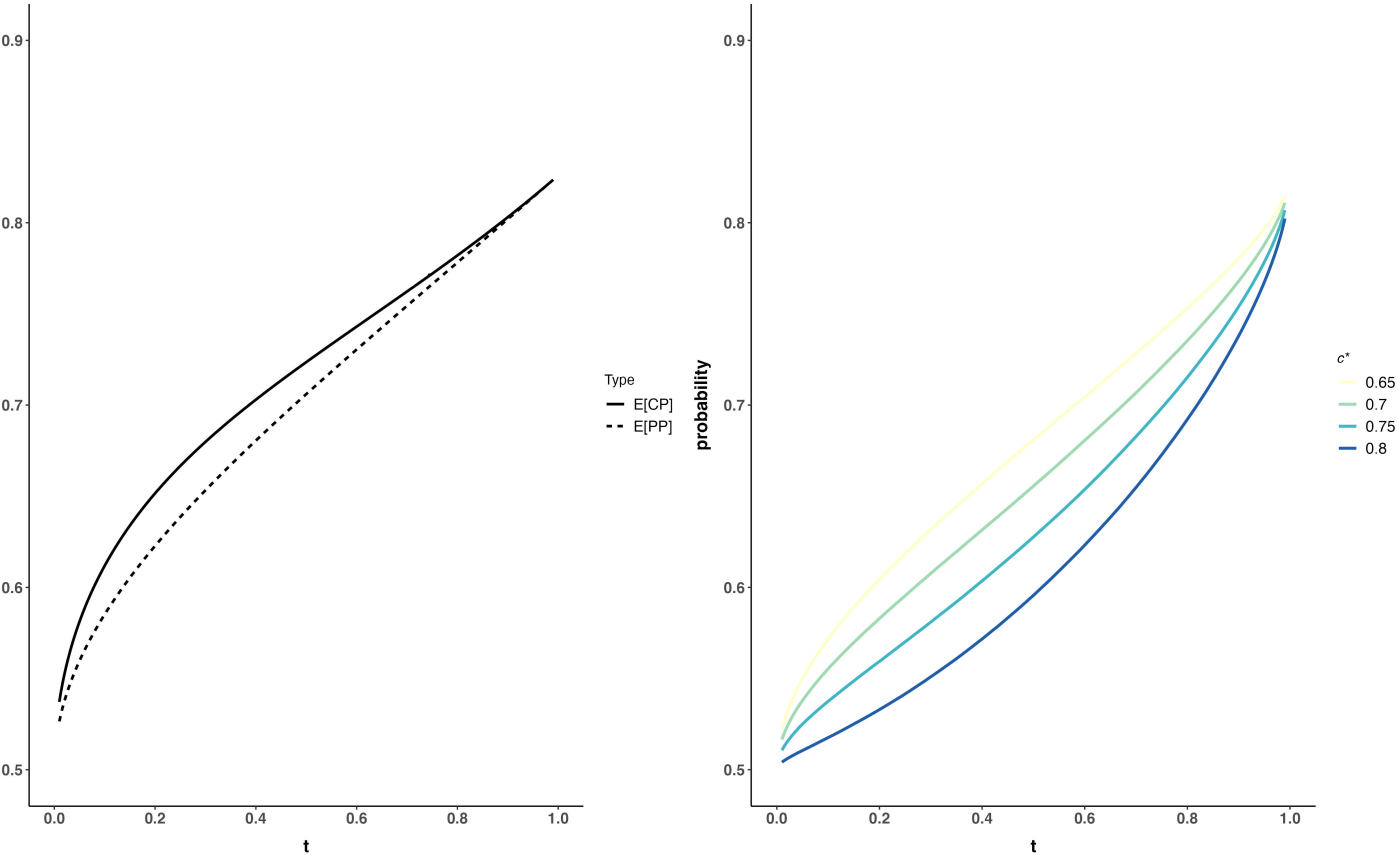
Power according to the timing of the interim analysis (t). On the left: E[CP] and E[PP] for a two‐arm clinical trial, binary endpoint, type I error of 0.025, power of 0.8, and true response rates associated to intervention and control arms $\pi_{I}=0.7$ and $\pi_{C}=0.4$. On the right: the probability of the conditional power being higher than the threshold $c^{*}$.

Figure 2 shows the trends of Equations (4), (5), (6) and (7) for a two‐arm clinical trial with binary endpoint, type I error of 0.025, power of 0.8, and true response rates associated to intervention and control arms $\pi_{I}=0.7$ and $\pi_{C}=0.4$, respectively. Analytical solutions may not be available, but the integrals in the previous formulas can be approximated using numerical approximation methods or Monte Carlo techniques. The corresponding functions are provided in the R script in the Supporting Information.

### 2.2 Augmented Analysis of the Clinical Trial and RWD

As explained in the introduction of this section, RWD may also be collected during the second stage of the trial if conditional approval has been granted. RWD call for careful analysis with dedicated techniques [19, 20] beyond the scope of this article. Including the right confounding variables and proper study design is crucial [21]. Analyzing the RWD to augment the clinical trial fits naturally in the target trial framework [22], which facilitates the drawing of valid conclusions in observational studies. In particular, the study design of the observational study should be chosen to match that of the clinical trial and RWD collected should be in the same format as the clinical trial data. However, a full description of RWD analyses fall outside the scope of this work and we refer the reader to the appropriate bibliography [19, 20].

To perform the final augmented analysis, when possible, we use the unsymmetrical meta‐analysis, suggested by Röver and Friede [23], via a variation of the well‐known normal‐normal hierarchical model (NNHM).

#### 2.2.1 The Normal‐Normal Hierarchical Model

The NNHM is a standard Bayesian framework used in meta‐analysis for continuous outcomes to account for both within‐ and between‐study variability. Let $g^{\left(i\right)}$ denote the observed value of the estimator $G^{\left(i\right)}$ from study i, with corresponding known variance ${\left(\sigma^{\left(i\right)}\right)}^{2}$. The model assumes

$$g^{\left(i\right)}|\theta^{\left(i\right)}∼𝒩(\theta^{\left(i\right)},{\left(\sigma^{\left(i\right)}\right)}^{2}),$$

where $\theta^{\left(i\right)}$ represents the true underlying effect in study i. These true effects are in turn assumed to follow a common distribution,

$$\theta^{\left(i\right)}|\mu ,\tau^{2}∼𝒩(\mu ,\tau^{2}),$$

where μ is the overall effect and $\tau^{2}$ is the between‐study variance, reflecting heterogeneity across studies. This two‐level hierarchical structure allows for partial pooling, borrowing strength across studies in a principled way. The model is typically completed by specifying prior distributions for the hyperparameters μ and τ, enabling full Bayesian inference.

#### 2.2.2 The Reference Model

In our situation, we aim at obtaining a shrinkage estimate for the effect of a specific study, the randomized one in our specific case, using the observational data, the RWD. Since it might seem counterintuitive to assume that both data refer to the same mean parameter or share identical variances, treating the estimates asymmetrically, by designating one as a “reference” estimate and the other as a “secondary” observable, with an uncertain degree of deviation, may be more appropriate. Following the parametrization suggested by Röver and Friede [23], we obtain

$$g^{\left(i\right)}|\theta^{\left(i\right)}∼𝒩\left(\theta^{\left(i\right)},{\left(\sigma^{\left(i\right)}\right)}^{2}\right),$$

for i∈{1,2}, where i=1 refers to the clinical trial results and i=2 refers to the RWD results. At the next level of the hierarchy, we specify

$$\theta^{\left(1\right)}|a,b∼𝒩\left(a,0\right)\;\text{and}\;\theta^{\left(2\right)}|a,b∼𝒩\left(a,b^{2}\right)$$

where the “effect” parameter a is assigned an improper uniform prior, and the variance component b is now assigned a prior density given by

$$\frac{1}{\sqrt{2}}p_{*}\left(\frac{b}{\sqrt{2}}\right),$$

where $p_{*}$ denotes the prior distribution that would be chosen for the heterogeneity parameter τ in a usual NNHM (see Röver and Friede [24], for more information on the usual NNHM parametrization and on τ). The first observable $g^{\left(1\right)}$ directly measures the parameter a (the reference), whereas the second observable $g^{\left(2\right)}$ incorporates an additional bias with variance. The variance component b once again accounts for the heterogeneity between the first and second observable, but in a manner that slightly differs from that in the original NNHM. The resulting ${\hat{\theta }}^{\left(1\right)}$ (or a in this specific case) represents the shrinkage estimation of the clinical trial, that is, an updated estimate for the study effect in the clinical trial, which is informed by the RWD. This final augmented analysis is easy to implement and the corresponding functions are provided in the R script in the Supporting Information, based on the original article [23].

## 3 Simulation Settings

We conducted a simulation study to evaluate the proposed framework, choosing as an example a binary endpoint study design. A summary of the notations used for the simulation study is presented in Table 2. For the clinical trial population, the true probability value associated to the intervention arm, $\pi_{I}^{\text{trial}}$, varies among {0.7,0.8,0.9}, and the true probability value associated to the control arm among $\left\{\pi_{I}^{\text{trial}}-0.3,\pi_{I}^{\text{trial}}-0.2,\pi_{I}^{\text{trial}}-0.1\right\}$. The total sample size n for the clinical trial is calculated from $\pi_{I}^{\text{trial}}$ and $\pi_{C}^{\text{trial}}$ so as to obtain a significance level equal to α=0.025 and a power equal to 1−β=0.9 for a one‐sided two‐sample test for proportions. Then, Equations (4) and (5) are computed with $\theta_{A}=\pi_{I}^{\text{trial}}-\pi_{C}^{\text{trial}}$, for values of the information time t in {0.5,0.6,0.7,0.8,0.9}. Finally, only scenarios for which E[CP] and E[PP] are closest to the target values {0.8,0.85,0.9} are retained.

**TABLE 2:** Summary of the notations used for the simulation study.

Notations	Meaning
For the clinical trial population	
$\pi_{I}^{\text{trial}}$, $\pi_{C}^{\text{trial}}$	True response rates associated to intervention and control arms
$\Delta^{\text{trial}}=\pi_{I}^{\text{trial}}-\pi_{C}^{\text{trial}}$	difference between the true response rates
At the end of the trial	
α	Type I error probability
β	Type II error probability
$n_{I}$, $n_{C}$	Number of individuals in the intervention and control arms
$n=n_{I}+n_{C}$	Total number of individuals
$x_{I}^{\text{trial}}$, $x_{C}^{\text{trial}}$	Observed binary intervention and control responses
$p_{I}^{\text{trial}}$, $p_{C}^{\text{trial}}$	Observed response rates in the intervention and control arms
$d^{\text{trial}}=p_{I}^{\text{trial}}-p_{C}^{\text{trial}}$	Difference between the observed response rates
At the interim time t∈(0,1)	
$n_{I,t}$, $n_{C,t}$	Number of individuals in the intervention and control arms
$n_{t}=n_{I,t}+n_{C,t}$	Total number of individuals
$x_{I,t}^{\text{trial}}$, $x_{C,t}^{\text{trial}}$	Observed binary intervention and control responses
$p_{I,t}^{\text{trial}}$, $p_{C,t}^{\text{trial}}$	Observed response rates in the intervention and control arms
$d_{t}^{\text{trial}}=p_{I,t}^{\text{trial}}-p_{C,t}^{\text{trial}}$	Difference between the observed response rates
For the RWD population	
$\pi_{I}^{\text{RWD}}$, $\pi_{C}^{\text{RWD}}$	True response rates associated to intervention and control arms
$\Delta^{\text{RWD}}=\pi_{I}^{\text{RWD}}-\pi_{C}^{\text{RWD}}$	Difference between the true response rates
In the RWD	
$m_{I}$, $m_{C}$	Number of individuals in the intervention and control arms
$m=m_{I}+m_{C}$	Total sample size
$d^{\text{RWD}}$	Difference between the observed response rates
$x_{I}^{\text{RWD}}$, $x_{C}^{\text{RWD}}$	Observed binary intervention and control responses
$p_{I}^{\text{RWD}}$, $p_{C}^{\text{RWD}}$	Observed response rates in the intervention and control arms

For the sake of simplicity, we assume that the allocation ratio (intervention arm to control arm) is 1:1 and there is no dropout. We also assume that the numbers of individuals in the intervention and control arms are equal to $n_{I}=n_{C}=n/2$, at the end of the trial, and to $n_{I,t}=n_{C,t}=n_{t}/2=tn/2$ at the time t of the interim analysis. The total sample size m of RWD (obtained in parallel with the second part of the trial thanks to the regulatory temporary authorization) is chosen equal to the number of individuals entering the clinical trial after time t, that is n(1−t), with equal number of individuals in the intervention and control arms $m_{I}=m_{C}=m/2$.

For the RWD population, the true probability value associated to the intervention arm, $\pi_{I}^{\text{RWD}}$, varies among $\left\{\pi_{I}^{\text{trial}}-0.6,\pi_{I}^{\text{trial}}\right\}$, and the true probability value associated to the control arm is chosen equal to $\pi_{C}^{\text{trial}}$. For the clinical trial (respectively, in the RWD), the binary intervention and control responses $x_{I}^{\text{trial}}$ and $x_{C}^{\text{trial}}$ (respectively, $x_{I}^{\text{RWD}}$ and $x_{C}^{\text{RWD}}$) are simulated using Bernoulli distributions with parameters $\pi_{I}^{\text{trial}}$ and $\pi_{C}^{\text{trial}}$ (respectively, $\pi_{I}^{\text{RWD}}$ and $\pi_{C}^{\text{RWD}}$, the response rates associated to intervention and control arms for the RWD population).

The values of the parameters used in the simulation study are summarized in Section 2 in the Supporting Information. For each scenario, 5000 data sets are simulated.

In the augmented analysis step, the primary interest is the shrinkage estimate of the study‐specific effect $\theta^{\left(1\right)}=\Delta^{\text{trial}}$ in the clinical trial population. The resulting precision is studied by comparing the 95% shrinkage interval width $\delta^{\text{trial}}$ with the original confidence interval width and by considering the *relative width* $q^{\text{trial}}=\delta^{\text{trial}}/(2\times 1.96\sigma^{\text{trial}})$. Assuming that standard errors scale with $1/\sqrt{n_{t}}$, the approximate gain in effective sample size can be estimated as ${\left(q^{\text{trial}}\right)}^{-2}-1$.

We use an improper uniform prior for μ and a half‐normal prior for the heterogeneity τ with scale 0.5. The induced priors on a and b are then derived as specified in Section 2.2.2.

All analyses are performed using R software version 4.3.0 using the packages *LongCart* version 3.2 and *bayesmeta* version 3.4.

## 4 Illustration of Two Simulated Data Sets

To illustrate the steps of our methodology, let us first focus on the results from two simulated data sets that differ in the true response rates associated to the intervention arm for the RWD. In this section, we use $\pi_{I}^{\text{trial}}=0.7$ and $\pi_{C}^{\text{trial}}=0.4$ as the true response rates for the clinical trial and $c^{*}=0.8$ as the threshold for the conditional power. In the first simulated data set, the true response rate associated to the intervention arm for the RWD is very different from that of the clinical trial: $\pi_{I}^{\text{RWD, 1}}=\pi_{I}^{\text{trial}}-0.6=0.1$. In contrast, in the second data set, the true response rates associated to the intervention arm for the RWD and clinical trial populations are equal: $\pi_{I}^{\text{RWD, 2}}=\pi_{I}^{\text{trial}}$. In both cases, the true response rates for the control arm for the RWD and clinical trial populations are equal: $\pi_{C}^{\text{RWD}}=\pi_{C}^{\text{trial}}$.

Using $\pi_{I}^{\text{trial}}$ and $\pi_{C}^{\text{trial}}$ as true response rates, the required total sample size n for the clinical trial is equal to 112 for a power equal to 0.9, with $n_{I}=n_{C}=56$ individuals each in the intervention and control arms. Then, the values $\theta_{A}=0.3$, $s_{n}^{2}=1/2[\pi_{I}^{\text{trial}}(1-\pi_{I}^{\text{trial}})+\pi_{C}^{\text{trial}}(1-\pi_{C}^{\text{trial}})]\approx 0.47^{2}$ and k≈0.09 are deduced and, from Equation (4), we obtain the following approximate values for the conditional power under the empirical trend averaged over the $Z_{t}$ distribution under the alternative hypothesis: {0.81,0.83,0.85,0.88,0.90} for t∈{0.5,0.6,0.7,0.8,0.9}. Thereafter, choosing the closest value to the threshold for the conditional power $c^{*}$ (i.e., 0.81), we deduce the timing of the interim analysis as t=0.5 and therefore the sample sizes $n_{I,t}=n_{C,t}=n_{t}/2=28$ for the intervention and control arms at interim analysis. In this example, the observed response rates at the end of the trial in the two arms are approximately equal to $p_{I}^{\text{trial}}\approx 0.70$ and $p_{C}^{\text{trial}}\approx 0.45$.

At the interim time t, the observed response rates in the two arms are equal to $p_{I,t}^{\text{trial}}\approx 0.71$ and $p_{C,t}^{\text{trial}}\approx 0.39$. Then, the conditional power CP is approximately equal to 0.99. Since $CP\geq c^{*}$, we can apply for regulatory temporary authorization.

At the end of the clinical trial, the null hypothesis that the proportions (probabilities of success) in the two arms are the same is tested with a confidence level equal to 1−α. The *p*‐value associated to the test is approximately equal to 0.004. Since it is less than 0.025, the test is significant and the null hypothesis is rejected.

**FIGURE 3:**
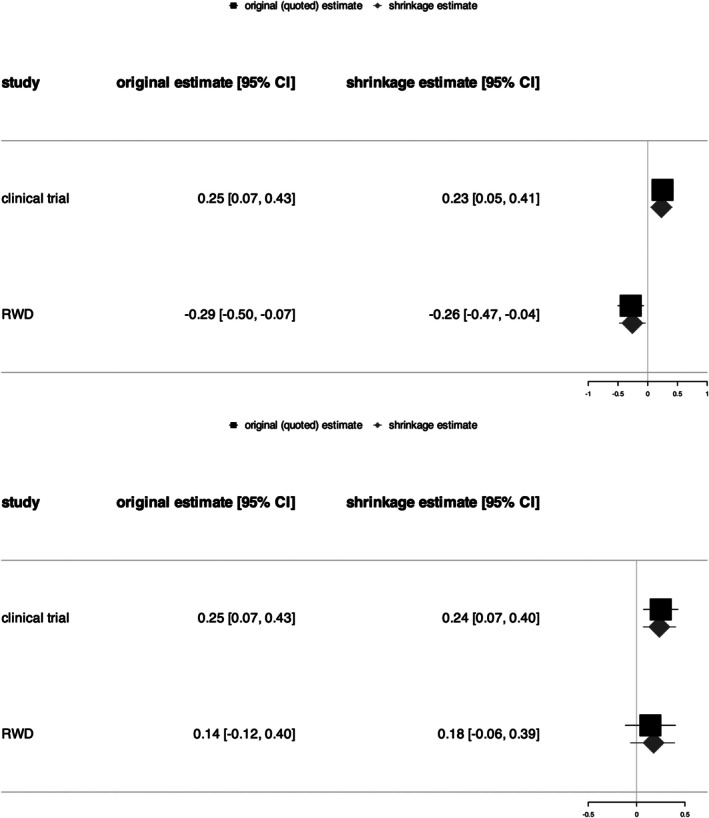
Forest plots for the illustration on two simulated data sets (risk difference outcome). On the top: when the true response rates associated to the intervention arm for the RWD and clinical trial populations are very different: $\pi_{I}^{\text{RWD, 1}}=\pi_{I}^{\text{trial}}-0.6=0.1$. On the bottom: when the true response rates associated to the intervention arm for the RWD and clinical trial populations are equal: $\pi_{I}^{\text{RWD, 2}}=\pi_{I}^{\text{trial}}$.

In parallel with the second part of the clinical trial, and thanks to the regulatory temporary authorization obtained following the interim analysis, a total sample size of RWD equal to the number of individuals entering the clinical trial after time t, that is m=n(1−t)=56, is available, with $m_{I}=m_{C}=28$ individuals in the intervention and control arms. The corresponding observed response rates in the two arms are approximately equal to $p_{I}^{\text{RWD, 1}}\approx 0.11$ and $p_{I}^{\text{RWD, 2}}\approx 0.54$ (for the two simulated data sets respectively), and $p_{C}^{\text{RWD}}\approx 0.39$. To perform the final augmented analysis using RWD, we use $g^{\left(1\right)}=d^{\text{trial}}=0.25$ and $\sigma^{\left(1\right)}=\sigma^{\text{trial}}\approx 0.09$, and, for each one of the two data sets, $g^{\left(2\right)}=d^{\text{RWD},1}\approx -0.29$ and $\sigma^{\left(2\right)}=\sigma^{\text{RWD},1}\approx 0.11$, or $g^{\left(2\right)}=d^{\text{RWD},2}\approx 0.14$ and $\sigma^{\left(2\right)}=\sigma^{\text{RWD},2}\approx 0.13$ (see Figure 3). Finally, the shrinkage estimates of the study‐specific effect in the clinical trial population (equal to $\Delta^{\text{trial}}=\pi_{I}^{\text{trial}}-\pi_{C}^{\text{trial}}=0.3$) for the two simulated data sets are very similar, ${\hat{\theta }}^{\left(1\right)}={\hat{\Delta }}^{\text{trial, 1}}=0.23$ and ${\hat{\theta }}^{\left(1\right)}={\hat{\Delta }}^{\text{trial, 2}}=0.24$, with gains in effective sample size approximately equal to −2% (that is a loss) and 12% respectively. This corresponds to increasing/reducing the sample size from the original sample size of n individuals to “effective number” of some n(1+gain), which means, in this example, from the sample size n=112 to approximately 110 and 125 for each simulated data set. To conclude this illustration, when the true response rate associated to the intervention arm for the RWD is very different from that of the clinical trial, the loss in effective sample size is low and the estimate is not much more biased than when the true response rates associated to the intervention arm for the RWD and clinical trial populations are equal. In this second case, there is a gain in effective sample size and therefore greater precision.

## 5 Simulation Results

This section gives only simulation results using CP, while the results for PP are shown in Section 3 of the Supporting Information. Moreover, only the cases when 60≤n≤300 are considered. The constraint of n≥60 ensures that there are at least 30 subjects per arm and the normal distribution can be used as an approximation in the meta‐analysis.

Table 3 gives a summary of the interim and final clinical trial results. In our scenarios, to obtain a threshold $c^{*}$ close to the values {0.8,0.85,0.9}, Equation (4) always gives the same corresponding values of the time t, that are {0.5,0.7,0.9}. As expected, for each scenario, the frequency when the *p*‐value of the final clinical trial analysis is lower than 0.025 is close to the power value of 0.9. Thus, the null hypothesis of equal response rates between intervention and control arms is mostly rejected. The CP value is greater than 0.8 in between 69% and 86% of cases, depending on the true response rates associated to the intervention and control arms for the clinical trial population (and consequently on n) and on t. Similarly, the CP value is greater than 0.85 (respectively 0.9) in between 66% and 85% of cases (respectively, between 60% and 83% of cases). In all these cases, the frequency when the *p*‐value is lower than 0.025 for the final clinical trial analysis is close to 1. Moreover, in these situations, the temporary regulatory authorization is obtained, and therefore RWD can be collected in parallel with the second part of the clinical trial.

**TABLE 3:** Interim and final clinical trial results for CP over 5000 replications.

$\pi_{I}^{\text{trial}}$	$\pi_{C}^{\text{trial}}$	n	t	CP	p≤α	CP≥0.8		CP≥0.85		CP≥0.9	
						Yes	p≤α	Yes	p ≤α	Yes	p≤α
0.7	0.4	112	0.5	0.80 (0.30)	0.89	0.70	0.97	0.69	0.97	0.60	0.98
0.7	0.4	112	0.7	0.84 (0.28)	0.89	0.78	0.98	0.73	0.98	0.70	0.99
0.7	0.4	112	0.9	0.88 (0.27)	0.89	0.83	0.99	0.83	0.99	0.83	0.99
0.7	0.5	248	0.5	0.80 (0.30)	0.90	0.71	0.97	0.66	0.98	0.63	0.98
0.7	0.5	248	0.7	0.84 (0.28)	0.90	0.77	0.98	0.73	0.99	0.69	0.99
0.7	0.5	248	0.9	0.88 (0.27)	0.90	0.82	0.99	0.82	0.99	0.79	1.00
0.8	0.5	104	0.5	0.81 (0.30)	0.91	0.70	0.98	0.68	0.98	0.66	0.98
0.8	0.5	104	0.7	0.85 (0.27)	0.91	0.77	0.99	0.74	0.99	0.72	0.99
0.8	0.5	104	0.9	0.89 (0.26)	0.91	0.84	0.99	0.83	0.99	0.80	1.00
0.8	0.6	218	0.5	0.79 (0.30)	0.90	0.69	0.98	0.66	0.98	0.62	0.98
0.8	0.6	218	0.7	0.84 (0.28)	0.90	0.75	0.99	0.72	0.99	0.68	0.99
0.8	0.6	218	0.9	0.88 (0.27)	0.90	0.84	0.99	0.82	1.00	0.81	1.00
0.9	0.6	84	0.5	0.83 (0.28)	0.92	0.76	0.98	0.71	0.98	0.65	0.98
0.9	0.6	84	0.7	0.86 (0.26)	0.92	0.79	0.99	0.77	0.99	0.73	0.99
0.9	0.6	84	0.9	0.90 (0.25)	0.92	0.86	0.99	0.85	0.99	0.82	0.99
0.9	0.7	164	0.5	0.81 (0.30)	0.90	0.70	0.98	0.68	0.98	0.65	0.99
0.9	0.7	164	0.7	0.85 (0.27)	0.90	0.77	0.99	0.74	0.99	0.70	0.99
0.9	0.7	164	0.9	0.89 (0.26)	0.90	0.85	0.99	0.83	0.99	0.81	0.99

*Note:* Mean and standard deviation of CP value, frequency (on all the 5000 replications) when the *p*‐value is lower than α=0.025 for the final analysis, frequencies when the CP value is greater than 0.8, 0.85, and 0.9 and frequencies (only for the corresponding replications) when the *p*‐value is lower than α=0.025 for the final analysis. CP: conditional power; $\pi_{I}^{\text{trial}}$, $\pi_{C}^{\text{trial}}$: true response rates associated to intervention and control arms for the clinical trial population; n: total number of individuals in the trial; t: information time; p: p‐value for the final analysis.

Table 4 gives the meta‐analysis results. The gain in precision may approximately be translated to an equivalent gain in effective sample size (as expressed through the $q^{\text{trial}}$ introduced above). Although this relative gain in information is always close to 0, it is slightly larger when data from clinical trials and RWD are similar (between 3% and 11%). As expected, the gain is greater if the sample size of RWD is large.

**TABLE 4:** Meta‐analysis results for CP over 5000 replications.

$\pi_{I}^{\text{trial}}$	$\pi_{C}^{\text{trial}}$	n	t	$\pi_{I}^{\text{RWD}}$	$\pi_{C}^{\text{RWD}}$	m	$d^{\text{trial}}$	${\hat{\Delta }}^{\text{trial}}$	$B(d^{\text{trial}})$	$B({\hat{\Delta }}^{\text{trial}})$	gain (%)
0.7	0.4	112	0.5	0.1	0.4	56	0.30	0.28	−0.00	−0.02	−1
0.7	0.4	112	0.7	0.1	0.4	34	0.30	0.28	−0.00	−0.02	−1
0.7	0.4	112	0.9	0.1	0.4	11	0.30	0.28	−0.00	−0.02	1
0.7	0.4	112	0.5	0.7	0.4	56	0.30	0.30	−0.00	−0.00	11
0.7	0.4	112	0.7	0.7	0.4	34	0.30	0.30	−0.00	−0.00	8
0.7	0.4	112	0.9	0.7	0.4	11	0.30	0.30	−0.00	−0.00	4
0.7	0.5	248	0.5	0.1	0.5	124	0.20	0.19	−0.00	−0.01	−1
0.7	0.5	248	0.7	0.1	0.5	74	0.20	0.19	−0.00	−0.01	−1
0.7	0.5	248	0.9	0.1	0.5	25	0.20	0.19	−0.00	−0.01	0
0.7	0.5	248	0.5	0.7	0.5	124	0.20	0.20	−0.00	−0.00	9
0.7	0.5	248	0.7	0.7	0.5	74	0.20	0.20	−0.00	−0.00	6
0.7	0.5	248	0.9	0.7	0.5	25	0.20	0.20	−0.00	−0.00	3
0.8	0.5	104	0.5	0.2	0.5	52	0.30	0.28	−0.00	−0.02	−1
0.8	0.5	104	0.7	0.2	0.5	31	0.30	0.28	−0.00	−0.02	−1
0.8	0.5	104	0.9	0.2	0.5	10	0.30	0.28	−0.00	−0.02	1
0.8	0.5	104	0.5	0.8	0.5	52	0.30	0.30	−0.00	−0.00	11
0.8	0.5	104	0.7	0.8	0.5	31	0.30	0.30	−0.00	−0.00	8
0.8	0.5	104	0.9	0.8	0.5	10	0.30	0.30	−0.00	−0.00	4
0.8	0.6	218	0.5	0.2	0.6	109	0.20	0.19	−0.00	−0.01	−1
0.8	0.6	218	0.7	0.2	0.6	65	0.20	0.19	−0.00	−0.01	−1
0.8	0.6	218	0.9	0.2	0.6	22	0.20	0.19	−0.00	−0.01	0
0.8	0.6	218	0.5	0.8	0.6	109	0.20	0.20	−0.00	−0.00	9
0.8	0.6	218	0.7	0.8	0.6	65	0.20	0.20	−0.00	−0.00	6
0.8	0.6	218	0.9	0.8	0.6	22	0.20	0.20	−0.00	−0.00	3
0.9	0.6	84	0.5	0.3	0.6	42	0.30	0.28	0.00	−0.02	−1
0.9	0.6	84	0.7	0.3	0.6	25	0.30	0.28	0.00	−0.02	0
0.9	0.6	84	0.9	0.3	0.6	8	0.30	0.29	0.00	−0.01	1
0.9	0.6	84	0.5	0.9	0.6	42	0.30	0.30	0.00	0.00	11
0.9	0.6	84	0.7	0.9	0.6	25	0.30	0.30	0.00	0.00	8
0.9	0.6	84	0.9	0.9	0.6	8	0.30	0.30	0.00	−0.00	4
0.9	0.7	164	0.5	0.3	0.7	82	0.20	0.19	−0.00	−0.01	−1
0.9	0.7	164	0.7	0.3	0.7	49	0.20	0.19	−0.00	−0.01	−1
0.9	0.7	164	0.9	0.3	0.7	16	0.20	0.19	−0.00	−0.01	0
0.9	0.7	164	0.5	0.9	0.7	82	0.20	0.20	−0.00	−0.00	9
0.9	0.7	164	0.7	0.9	0.7	49	0.20	0.20	−0.00	−0.00	7
0.9	0.7	164	0.9	0.9	0.7	16	0.20	0.20	−0.00	−0.00	3

*Note:* Mean difference between the observed response rates at the end of the trial ($d^{\text{trial}}$) and corresponding bias ($B(d^{\text{trial}})$), mean estimated difference between the response rates after meta‐analysis (${\hat{\Delta }}^{\text{trial}}$) and corresponding bias $B({\hat{\Delta }}^{\text{trial}})$, and mean proportion of gain (%) in effective sample size when using the shrinkage estimate relative to the original “plain” CI (gain). The gain in effective sample size is approximated as ${\left(q^{\text{trial}}\right)}^{-2}-1$ where $q^{\text{trial}}=\delta^{\text{trial}}/(2\times 1.96\sigma^{\text{trial}})$ is the *relative width* and $\delta^{\text{trial}}$ the 95% shrinkage interval width. CP: conditional power; CI: confidence interval; $\pi_{I}^{\text{trial}}$, $\pi_{C}^{\text{trial}}$: true response rates associated to intervention and control arms for the clinical trial population; n: total number of individuals in the trial; t: information time; $\pi_{I}^{\text{RWD}}$, $\pi_{C}^{\text{RWD}}$: true response rates associated to intervention and control arms for the RWD population; m: total sample size of the RWD population.

The results for PP, given in the Supporting Information, do not differ substantially from those obtained for CP. Additional simulation results, including either other values of the true response rates associated to intervention ($\pi_{I}^{\text{trial}}$, $\pi_{I}^{\text{RWD}}$) and control arms ($\pi_{C}^{\text{trial}}$, $\pi_{C}^{\text{RWD}}$) in the trial and RWD populations, or a threshold $c^{*}\in \{0.6,0.7,0.8\}$ and time t∈{0.1,0.2,0.3,0.4,0.5,0.6}, are given in Section 4 in the Supporting Information. The Supporting Information also includes, in Section 5, a realistic example built on the AXOMOVE THERAPY case study.

## 6 Conclusion

The European fast‐track requirements impose new evaluation processes for DMDs. We propose a clinical trials framework that enables a more rigorous evaluation under these requirements. In particular, several metrics are proposed to determine the appropriate time for interim analysis and to apply for regulatory temporary authorization. We further recommend adhering to common practice by evaluating simulations conducted under the null hypothesis (see Supporting Information, Section 4) to determine whether the chosen timing of the interim analysis meets acceptable error levels for temporary approval in cases where the final analysis is negative. Indeed, in this case and under the null hypothesis, a device would reach more patients while it should not have, so it would do some harm. Maintaining safe and efficient access to innovation is mandatory while allowing patients to benefit from innovations in a timely manner. For clinical trial stakeholders, methodologists and regulators, these two requirements are not always compatible. For these reasons, we have proposed a design that accounts for these two conditions but which is still based on rigorous statistical properties. While the statistical framework is presented using only one interim analysis in this article, it can be integrated with other more complex study designs, such as group‐sequential design. In a group‐sequential design, several interim analyses are planned to determine whether to continue the trial. If necessary, our framework can be adapted to function as an overlay to the existing trial design, and to not alter the core structure or methodology of the trial. Consequently, it will not impact the type I error rate or the statistical power of the study. This compatibility allows for enhanced flexibility and robustness in the trial without compromising the integrity of the statistical analysis.

However, fast‐track authorization following interim analysis could also have several negative impacts on the clinical trial. For example, randomizing patients to the control group without proposing use of the device to them could raise ethical concerns. Furthermore, a contamination bias or a differential drop‐out in the clinical trial could also arise if enrolled individuals in the control group use the DMD. One way of setting out to correct this is to propose DMDs to individuals in the control group at the end of the study. This is known as a waiting list. This is done when there is a fear that inclusions will be limited due to a lack of motivation to form part of the control group. Nevertheless, the fast‐track authorization is already in place, although its impact is beyond the scope of this article.

In our framework, RWD is collected by the manufacturer of the DMD (thanks to the regulatory temporary authorization) in parallel with the second part of the trial. In view of the reimbursement request, it can be logically assumed that this collection was initially foreseen in the protocol and that RWD is collected in the same format as clinical trial data to facilitate their analysis. As a result, there is a single source of RWD. In any case, if reimbursement authorization is granted for the DMD, RWD will be available in administrative databases. For instance, in France, the SNDS (*Système National des Données de Santé*) is a warehouse of pseudonymized medico‐administrative data covering the entire French population, and containing all care submitted for reimbursement. However, the issue of multiple RWD sources falls outside the scope of this work but could be addressed in future research.

Combining RWD and RCT can serve multiple aims [13, 25], such as improving generalizability or transportability [13] (translating trial findings to real‐world settings), augmenting control arms [26] (leveraging RWD for RCTs), debiasing [27] (correcting hidden confounding in observational data), or integrating results across sources. For the latter, most approaches adopt a standard meta‐analysis framework, thoroughly assessing observational estimates to detect potential biases [28]. Alternative techniques include shrinkage estimation [29] or the test‐then‐merge paradigm [30], which first evaluates the similarity between RWD and RCT outcomes before applying a robust integration method. We selected the Bayesian implementation of the NNHM for its interpretability and ease of implementation. Nevertheless, the aforementioned methods remain valuable, particularly when a preliminary causal‐inference analysis of RWD is warranted.

Incorporating RWD in our approach enhances the precision of the final effect estimate compared to the standard approach. Nevertheless, the simulation study showed that the gains in effective sample size were relatively small in some scenarios. These results depend on assumptions such as that the RWD sample size is equal to the number of individuals entering the clinical trial after the interim time. In real life, the DMD could be more widely distributed in the population, thereby increasing the corresponding sample size and, consequently, the gain. In any case, the authorities are calling for RWD to be collected and analyzed, as these bring heterogeneity in terms of patient type and results closer to real‐world practice, thus allowing higher external validity. This framework should come with a post‐market evaluation of the DMD once it is broadly used in the spirit of phase IV studies. It is important to note that the final augmented analysis is intended to refine the clinical trial results. However, it can also be used in the opposite direction as a first assessment of the generalizability of the trial results. When patient characteristics differ substantially between the clinical trial and RWD, discrepancies in treatment effects may arise. In such instances, methods aimed at improving generalizability or transportability, such as weighting, stratification, or matching [12, 13], can be applied to assess whether the trial findings remain applicable. However, this problem is beyond the scope of our study, and interested readers may refer to the works of Efthimiou et al. [31] and Dahabreh et al. [32]. Nevertheless, if significant differences in effect estimates persist, the final meta‐analysis will typically reveal this through observed heterogeneity. Importantly, the original trial results will remain largely unaffected, as demonstrated in the simulation study. In these situations, the sponsor can further explore sources of disagreement before submitting the findings to regulatory authorities. When making final decisions, HTA bodies must also take account of type I error inflation due to the consideration of two analyses (final clinical trial analysis and augmented analysis with RWD). If no heterogeneity is observed, it suggests that the clinical trial results may be applicable to the broader population. Nevertheless, deeper analyses may be necessary to confirm this reflection and ensure comprehensive understanding. Indeed, if the main objective of the clinical trial is the evaluation of clinical efficacy, effectiveness in real‐life conditions matters greatly. DMDs should be considered as part of complex systems and thus fall within the scope of complex intervention evaluation. Beyond the clinical effectiveness of the tool, its real‐world use, its organizational impact as well as its perceived efficacy could represent new endpoints. Evaluating this type of outcome takes even longer, and requires other study schemes.

In the last two years, DMDs, whether standalone or associated to drug administration, have radically changed the way patients are taken care of. For instance, cancer patients are prescribed telemonitoring‐based mobile apps, in which they share their data, including biology, adverse events, clinical endpoints and quality of life. Sometimes these app are interoperable with other medical devices as a means of cardiovascular or blood sugar level monitoring. These data are shared via the device with their physician and hospital which, after analysis, enable a better personalization of treatment and avoid major adverse events in between visits to their health care professional. The way these devices are evaluated is still via statistically based clinical study designs. However, these methods should be adapted to its use and implemented in the population. Indeed, in the future, the digital device should not be evaluated as a drug, but as a complex care intervention with multiple components, often integrating many different users in constant interaction, mobilizing specific behaviors or even specific skills on the part of these users to use the device. Before their effectiveness can be assessed, digital devices must have been the subject of pilot studies to study the feasibility of their deployment and evaluation. When it comes to assessing the effect of the device, it is essential to analyze both the use of the device and the conditions under which it is used. This information is crucial for the generalization of results and the deployment of digital devices [33]. Tandon et al. [34] specify key points for evaluating the effect of digital devices: “(1) consider an in‐depth assessment of technology usability beyond user satisfaction and ease of use, (2) expand recruitment to include important user groups such as clinicians and care partners, (3) report the rationale for key study design considerations including the sample size, and (4) provide rich descriptive statistics regarding the study sample to allow a complete understanding of generalizability to other patient populations and contexts of use.”

This opens a new field for clinical trials designs development, as these DMDs are associated with a number of specificities which are different from a drug that does not evolve over time. The statistical methodologist community should put forward proper design accounting to these health technology innovations which are radically transforming approaches to patient care and the monitoring of health trajectories.

## Funding

This work was supported by the Agence Nationale de la Recherche (Grant No. ANR‐22‐PESN‐0003).

## Conflicts of Interest

The authors declare no conflicts of interest.

## Supporting information




**Data S1.** Supporting Information.

## Data Availability

Data sharing not applicable to this article as no datasets were generated or analysed during the current study.
