# Supplementary material for: Innovative Clinical Trial Approach for Evaluating Digital Medical Devices Under European Fast‐Track Regulatory Frameworks
Source: Stat Med. 2026 May 5;45:e70572. doi: 10.1002/sim.70572 (PMC13143562; doi:10.1002/sim.70572)
Supplement: Supplementary file 1 — Data S1. Supporting Information. [file SIM-45-0-s001.zip › Ursino_2025_supporting_information_review2.pdf]

Innovative Clinical Trial Approach for Evaluating Digital Medical Devices under European Fast-Track Regulatory Frameworks

Supplementary Material

1 Derivation of conditional power and predictive power

Lan et al. (2009) considered the z-score Z^{Lan} at the end of the trial that is approximately normally distributed with unit variance and means 0 and θ^{Lan} under the null and alternative hypotheses. Using the notation of our manuscript, $\theta^{\text{Lan}} = \theta/k$, where k denote the standard deviation of the estimator. $Z^{\text{Lan}}(t)$, equivalent to our Z_t, is the z-statistic at information time t, so that $Z^{\text{Lan}}(1)$ is the z-score at the end of the trial. The mean of $Z^{\text{Lan}}(t)$ is equal to $\theta^{\text{Lan}} t^{1/2}$, where θ^{Lan} is the expected z-statistic at the end of the trial.

The authors also introduced the B-value, $B^{\text{Lan}}(t)$, defined by $B^{\text{Lan}}(0) = 0$ and $B^{\text{Lan}}(t) = t^{1/2} Z^{\text{Lan}}(t)$ for $t > 0$ to demonstrate that the conditional power is equal to

$$\text{CP} = \Phi\left(\frac{b^{\text{Lan}} + \theta^{\text{Lan}}(1-t) - z^{\text{Lan}}_{\alpha^{\text{Lan}}/2}}{\sqrt{1-t}}\right),$$

where $b^{\text{Lan}} = \sqrt{t} z_t$ is the observed value of $B^{\text{Lan}}(t)$, $\theta^{\text{Lan}} = \theta'/k$ and $z^{\text{Lan}}_{\alpha^{\text{Lan}}/2} = z_{1-\alpha}$. We have chosen to write θ' instead of θ to highlight that the value of this variable can be chosen when computing the CP or PP.

The authors also proved in their equation 5 that the predictive power is equal to:

$$\text{PP(t)} = \Phi\left\{\frac{(\hat{\theta}^{\text{Lan}}_e - z^{\text{Lan}}_{\alpha^{\text{Lan}}/2})\sqrt{t}}{\sqrt{1-t}}\right\},$$

that can be rewritten:

$$\text{PP(t)} = \Phi\left\{\frac{\sqrt{t}(\hat{\theta}^{\text{Lan}}_e t^{1/2}) - t z^{\text{Lan}}_{\alpha^{\text{Lan}}/2}}{\sqrt{t(1-t)}}\right\},$$

where $\hat{\theta}^{\text{Lan}}_e$ is the empirical trend.

2 Complement to simulation settings

The values of the parameters used in the simulation study in the main manuscript are given in Table 1.

Table 1: Parameter values in the simulation study.

**Parameter**	**Values**
α	0.025
$1-\beta$	0.9
π_I^{trial}	0.7, 0.8, 0.9
π_C^{trial}	$\pi_I^{\text{trial}} - 0.3, \pi_I^{\text{trial}} - 0.2, \pi_I^{\text{trial}} - 0.1$
π_I^{RWD}	$\pi_I^{\text{trial}} - 0.6$, π_I^{trial}
π_C^{RWD}	π_C^{trial}
t	0.5, 0.6, 0.7, 0.8, 0.9
c^*	0.8, 0.85, 0.9
m	$\text{round}(n \times (1-t))$
m_I, m_C	$m/2$

3 Simulation results for predictive power

Tables 2 and 3 show the results when the predictive power (PP) is used as metric to define the timing of the interim analysis.

As expected, for each scenario, the frequency when the p-value of the final clinical trial analysis is lower than 0.025 is close to the power value of 0.9 when $t = 0.9$. Thus, the null hypothesis of equal response rates between intervention and control arms is mostly rejected. The PP value is greater than 0.8 in between 61% and 86% of cases, depending on the true responses rates associated to the intervention and control arms for the clinical trial population (and, consequently, on n) and on t. Similarly, the PP value is greater than 0.85 (respectively 0.9) in between 60% and 85% of cases (respectively, between 50% and 82% of cases). In all these cases, the frequency when the p-value is lower than 0.025 for the final clinical trial analysis is close to 1. Moreover, in these situations, the temporary regulatory authorization is obtained, and therefore real-world data (RWD) can be collected in parallel with the second part of the clinical trial.

Table 2: Interim and final clinical trial results for PP over 5,000 replications. Mean and standard deviation of PP value, frequency (on all the 5,000 replications) when the p-value is lower than $\alpha = 0.025$ for the final analysis, frequencies when the PP value is greater than 0.8, 0.85 and 0.9 and frequencies (only for the corresponding replications) when the p-value is lower than $\alpha = 0.025$ for the final analysis. PP: predictive power; π_I^{trial}, π_C^{trial}: true response rates associated to intervention and control arms for the clinical trial population; n: total number of individuals in the trial; t: information time; pval: p-value for the final analysis.

						**PP ≥ 0.8**		**PP ≥ 0.85**		**PP ≥ 0.9**	
π_I^{trial}	π_C^{trial}	**n**	**t**	**PP**	**pval ≤ α**	**Yes**	**pval ≤ α**	**Yes**	**pval ≤ α**	**Yes**	**pval ≤ α**
0.7	0.4	112	0.5	0.78 (0.28)	0.89	0.61	0.98	0.60	0.98	0.50	0.99
0.7	0.4	112	0.7	0.84 (0.27)	0.89	0.75	0.98	0.70	0.99	0.70	0.99
0.7	0.4	112	0.9	0.88 (0.27)	0.89	0.83	0.99	0.83	0.99	0.80	0.99
0.7	0.5	248	0.6	0.81 (0.27)	0.90	0.69	0.98	0.66	0.99	0.59	0.99
0.7	0.5	248	0.8	0.85 (0.27)	0.90	0.78	0.99	0.75	0.99	0.72	1.00
0.7	0.5	248	0.9	0.88 (0.27)	0.90	0.82	0.99	0.81	0.99	0.79	1.00
0.8	0.5	104	0.5	0.79 (0.27)	0.91	0.66	0.98	0.60	0.99	0.55	0.99
0.8	0.5	104	0.7	0.84 (0.26)	0.91	0.75	0.99	0.73	0.99	0.67	0.99
0.8	0.5	104	0.9	0.89 (0.26)	0.91	0.84	0.99	0.83	0.99	0.80	1.00
0.8	0.6	218	0.6	0.80 (0.27)	0.90	0.69	0.98	0.64	0.99	0.58	0.99
0.8	0.6	218	0.7	0.83 (0.27)	0.90	0.73	0.99	0.69	0.99	0.64	0.99
0.8	0.6	218	0.9	0.88 (0.27)	0.90	0.83	0.99	0.82	1.00	0.81	1.00
0.9	0.6	84	0.5	0.81 (0.26)	0.92	0.66	0.98	0.64	0.99	0.58	0.99
0.9	0.6	84	0.7	0.85 (0.25)	0.92	0.77	0.99	0.73	0.99	0.70	0.99
0.9	0.6	84	0.9	0.90 (0.24)	0.92	0.86	0.99	0.85	0.99	0.82	0.99
0.9	0.7	164	0.6	0.81 (0.27)	0.90	0.69	0.99	0.66	0.99	0.61	0.99
0.9	0.7	164	0.7	0.84 (0.26)	0.90	0.74	0.99	0.72	0.99	0.67	0.99
0.9	0.7	164	0.9	0.89 (0.26)	0.90	0.85	0.99	0.83	0.99	0.80	1.00

The gain in precision may approximately be translated to an equivalent gain in effective sample size (as expressed through the q^{trial} introduced in the main manuscript). Although this relative gain in information is always close to 0, it is slightly larger when data from clinical trials and RWD are similar (between 3% and 11 %). As expected, the gain is greater if the sample size of RWD is large.

Table 3: Meta-analysis results for PP over 5,000 replications. Mean difference between the observed response rates at the end of the trial (d^{trial}) and corresponding bias ($B(d^{\text{trial}})$), mean estimated difference between the response rates after meta-analysis ($\hat{\Delta}^{\text{trial}}$) and corresponding bias $B(\hat{\Delta}^{\text{trial}})$, and mean proportion of gain (%) in effective sample size when using the shrinkage estimate relative to the original "plain" CI (gain). The gain in effective sample size is approximated as $(q^{\text{trial}})^{-2} - 1$ where $q^{\text{trial}} = \delta^{\text{trial}}/(2 \times 1.96\sigma^{\text{trial}})$ is the *relative width* and δ^{trial} the 95% shrinkage interval width.
PP: predictive power; CI: confidence interval; π_I^{trial}, π_C^{trial}: true response rates associated to intervention and control arms for the clinical trial population; n: total number of individuals in the trial; t: information time; π_I^{RWD}, π_C^{RWD}: true response rates associated to intervention and control arms for the RWD population; m: total sample size of the RWD population.

$\pi_{\mathbf{I}}^{\text{trial}}$	$\pi_{\mathbf{C}}^{\text{trial}}$	**n**	**t**	$\pi_{\mathbf{I}}^{\text{RWD}}$	$\pi_{\mathbf{C}}^{\text{RWD}}$	**m**	$\mathbf{d}^{\text{trial}}$	$\hat{\mathbf{\Delta}}^{\text{trial}}$	$\mathbf{B}(\mathbf{d}^{\text{trial}})$	$\mathbf{B}(\hat{\mathbf{\Delta}}^{\text{trial}})$	**gain (%)**
0.7	0.4	112	0.5	0.1	0.4	56	0.30	0.28	-0.00	-0.02	-1
0.7	0.4	112	0.7	0.1	0.4	34	0.30	0.28	-0.00	-0.02	-1
0.7	0.4	112	0.9	0.1	0.4	11	0.30	0.28	-0.00	-0.02	1
0.7	0.4	112	0.5	0.7	0.4	56	0.30	0.30	-0.00	-0.00	11
0.7	0.4	112	0.7	0.7	0.4	34	0.30	0.30	-0.00	-0.00	8
0.7	0.4	112	0.9	0.7	0.4	11	0.30	0.30	-0.00	-0.00	4
0.7	0.5	248	0.6	0.1	0.5	99	0.20	0.19	-0.00	-0.01	-1
0.7	0.5	248	0.8	0.1	0.5	50	0.20	0.19	-0.00	-0.01	-1
0.7	0.5	248	0.9	0.1	0.5	25	0.20	0.19	-0.00	-0.01	0
0.7	0.5	248	0.6	0.7	0.5	99	0.20	0.20	-0.00	-0.00	8
0.7	0.5	248	0.8	0.7	0.5	50	0.20	0.20	-0.00	-0.00	5
0.7	0.5	248	0.9	0.7	0.5	25	0.20	0.20	-0.00	-0.00	3
0.8	0.5	104	0.5	0.2	0.5	52	0.30	0.28	-0.00	-0.02	-1
0.8	0.5	104	0.7	0.2	0.5	31	0.30	0.28	-0.00	-0.02	-1
0.8	0.5	104	0.9	0.2	0.5	10	0.30	0.28	-0.00	-0.02	1
0.8	0.5	104	0.5	0.8	0.5	52	0.30	0.30	-0.00	-0.00	11
0.8	0.5	104	0.7	0.8	0.5	31	0.30	0.30	-0.00	-0.00	8
0.8	0.5	104	0.9	0.8	0.5	10	0.30	0.30	-0.00	-0.00	4
0.8	0.6	218	0.6	0.2	0.6	87	0.20	0.19	-0.00	-0.01	-1
0.8	0.6	218	0.7	0.2	0.6	65	0.20	0.19	-0.00	-0.01	-1
0.8	0.6	218	0.9	0.2	0.6	22	0.20	0.19	-0.00	-0.01	0
0.8	0.6	218	0.6	0.8	0.6	87	0.20	0.20	-0.00	-0.00	8
0.8	0.6	218	0.7	0.8	0.6	65	0.20	0.20	-0.00	-0.00	6
0.8	0.6	218	0.9	0.8	0.6	22	0.20	0.20	-0.00	-0.00	3
0.9	0.6	84	0.5	0.3	0.6	42	0.30	0.28	0.00	-0.02	-1
0.9	0.6	84	0.7	0.3	0.6	25	0.30	0.28	0.00	-0.02	0
0.9	0.6	84	0.9	0.3	0.6	8	0.30	0.29	0.00	-0.01	1
0.9	0.6	84	0.5	0.9	0.6	42	0.30	0.30	0.00	0.00	11
0.9	0.6	84	0.7	0.9	0.6	25	0.30	0.30	0.00	0.00	8
0.9	0.6	84	0.9	0.9	0.6	8	0.30	0.30	0.00	-0.00	4
0.9	0.7	164	0.6	0.3	0.7	66	0.20	0.19	-0.00	-0.01	-1
0.9	0.7	164	0.7	0.3	0.7	49	0.20	0.19	-0.00	-0.01	-1
0.9	0.7	164	0.9	0.3	0.7	16	0.20	0.19	-0.00	-0.01	0
0.9	0.7	164	0.6	0.9	0.7	66	0.20	0.20	-0.00	-0.00	8
0.9	0.7	164	0.7	0.9	0.7	49	0.20	0.20	-0.00	-0.00	7
0.9	0.7	164	0.9	0.9	0.7	16	0.20	0.20	-0.00	-0.00	3

4 Complements to simulation results

Additional simulation results when the CP is used as metric to define the timing of the interim analysis are shown for $\pi_I^{\text{trial}} = 0.7$ in Table 4, for $\pi_I^{\text{trial}} = 0.8$ and $\pi_C^{\text{trial}} = 0.5$ in Table 5, for $\pi_I^{\text{trial}} = 0.8$ and $\pi_C^{\text{trial}} = 0.6$ in Table 6, for $\pi_I^{\text{trial}} = 0.9$ and $\pi_C^{\text{trial}} = 0.6$ in Table 7 and for $\pi_I^{\text{trial}} = 0.9$ and $\pi_C^{\text{trial}} = 0.7$ in Table 8:

- In Table 4, $\pi_I^{\text{trial}} = 0.7$ and π_I^{RWD} varies in $\{0.1, 0.3, 0.5, 0.7\}$, so the corresponding drifts between the clinical trial and RWD true responses rates associated to intervention arm are equal to $\{0.6, 0.4, 0.2, 0\}$. In the same way, when $\pi_C^{\text{trial}} = 0.4$ or $\pi_C^{\text{trial}} = 0.5$, π_C^{RWD} varies in $\{0.2, 0.4\}$, so the corresponding drifts between the clinical trial and RWD true responses rates associated to control arm are $\{0.2, 0\}$ or $\{0.3, 0.1\}$;
- In Table 5, $\pi_I^{\text{trial}} = 0.8$ and π_I^{RWD} varies in $\{0.2, 0.4, 0.6, 0.8\}$, so the corresponding drifts are equal to $\{0.6, 0.4, 0.2, 0\}$. In the same way, $\pi_C^{\text{trial}} = 0.5$ and π_C^{RWD} varies in $\{0.2, 0.4\}$, so the corresponding drifts are $\{0.3, 0.1\}$;
- In Table 6, $\pi_I^{\text{trial}} = 0.8$ and π_I^{RWD} varies in $\{0.2, 0.4, 0.6, 0.8\}$, so the corresponding drifts are equal to $\{0.6, 0.4, 0.2, 0\}$. In the same way, $\pi_C^{\text{trial}} = 0.6$ and π_C^{RWD} varies in $\{0.2, 0.4, 0.6\}$, so the corresponding drifts are $\{0.4, 0.2, 0\}$;
- In Table 7, $\pi_I^{\text{trial}} = 0.9$ and π_I^{RWD} varies in $\{0.1, 0.3, 0.5, 0.7, 0.9\}$, so the corresponding drifts are equal to $\{0.8, 0.6, 0.4, 0.2, 0\}$. In the same way, $\pi_C^{\text{trial}} = 0.6$ and π_C^{RWD} varies in $\{0.2, 0.4, 0.6\}$, so the corresponding drifts are $\{0.4, 0.2, 0\}$;
- In Table 8, $\pi_I^{\text{trial}} = 0.9$ and π_I^{RWD} varies in $\{0.1, 0.3, 0.5, 0.7, 0.9\}$, so the corresponding drifts are equal to $\{0.8, 0.6, 0.4, 0.2, 0\}$. In the same way, $\pi_C^{\text{trial}} = 0.7$ and π_C^{RWD} varies in $\{0.1, 0.3, 0.5, 0.7\}$, so the corresponding drifts are $\{0.6, 0.4, 0.2, 0\}$.

Tables 9, 10, 11 and 12 give additional simulation results including a threshold $c^* \in \{0.6, 0.7, 0.8\}$ and time $t \in \{0.1, 0.2, 0.3, 0.4, 0.5, 0.6\}$ while Table 13 provides additional simulation results when $\pi_I^{\text{trial}} = \pi_C^{\text{trial}}$.

Table 4: Complement to meta-analysis results for CP, when $\pi_I^{\text{trial}} = 0.7$, over 5,000 replications. Mean difference between the observed response rates at the end of the trial (d^{trial}) and corresponding bias ($B(d^{\text{trial}})$), mean estimated difference between the response rates after meta-analysis ($\hat{\Delta}^{\text{trial}}$) and corresponding bias $B(\hat{\Delta}^{\text{trial}})$, and mean proportion of gain (%) in effective sample size when using the shrinkage estimate relative to the original "plain" CI (gain). The gain in effective sample size is approximated as $(q^{\text{trial}})^{-2} - 1$ where $q^{\text{trial}} = \delta^{\text{trial}}/(2 \times 1.96\sigma^{\text{trial}})$ is the *relative width* and δ^{trial} the 95% shrinkage interval width.
CP: conditional power; CI: confidence interval; π_I^{trial}, π_C^{trial}: true response rates associated to intervention and control arms for the clinical trial population; n: total number of individuals in the trial; t: information time; π_I^{RWD}, π_C^{RWD}: true response rates associated to intervention and control arms for the RWD population; m: total sample size of the RWD population; $\text{drift}_I = \pi_I^{\text{trial}} - \pi_I^{\text{RWD}}$: drift between the clinical trial and RWD true responses rates associated to intervention arm; $\text{drift}_C = \pi_C^{\text{trial}} - \pi_C^{\text{RWD}}$: drift between the clinical trial and RWD true responses rates associated to control arm.

$(\pi_\mathbf{I}^{\text{trial}}, \pi_\mathbf{C}^{\text{trial}})$	n	t	$(\pi_\mathbf{I}^{\text{RWD}}, \pi_\mathbf{C}^{\text{RWD}})$	m	$(\text{drift}_I, \text{drift}_C)$	$\mathbf{d}^{\text{trial}}$	$\hat{\mathbf{\Delta}}^{\text{trial}}$	$\mathbf{B}(\mathbf{d}^{\text{trial}})$	$\mathbf{B}(\hat{\mathbf{\Delta}}^{\text{trial}})$	gain (%)
(0.7, 0.4)	112	0.5	(0.1, 0.2)	56	(0.6, 0.2)	0.30	0.28	-0.00	-0.02	-1
(0.7, 0.4)	112	0.7	(0.1, 0.2)	34	(0.6, 0.2)	0.30	0.28	-0.00	-0.02	-0
(0.7, 0.4)	112	0.9	(0.1, 0.2)	11	(0.6, 0.2)	0.30	0.28	-0.00	-0.02	2
(0.7, 0.4)	112	0.5	(0.3, 0.2)	56	(0.4, 0.2)	0.30	0.28	-0.00	-0.02	7
(0.7, 0.4)	112	0.7	(0.3, 0.2)	34	(0.4, 0.2)	0.30	0.28	-0.00	-0.02	6
(0.7, 0.4)	112	0.9	(0.3, 0.2)	11	(0.4, 0.2)	0.30	0.29	-0.00	-0.01	4
(0.7, 0.4)	112	0.5	(0.3, 0.4)	56	(0.4, 0.0)	0.30	0.28	-0.00	-0.02	0
(0.7, 0.4)	112	0.7	(0.3, 0.4)	34	(0.4, 0.0)	0.30	0.28	-0.00	-0.02	1
(0.7, 0.4)	112	0.9	(0.3, 0.4)	11	(0.4, 0.0)	0.30	0.29	-0.00	-0.01	2
(0.7, 0.4)	112	0.5	(0.5, 0.2)	56	(0.2, 0.2)	0.30	0.30	-0.00	-0.00	11
(0.7, 0.4)	112	0.7	(0.5, 0.2)	34	(0.2, 0.2)	0.30	0.30	-0.00	-0.00	8
(0.7, 0.4)	112	0.9	(0.5, 0.2)	11	(0.2, 0.2)	0.30	0.30	-0.00	-0.00	4
(0.7, 0.4)	112	0.5	(0.5, 0.4)	56	(0.2, 0.0)	0.30	0.28	-0.00	-0.02	6
(0.7, 0.4)	112	0.7	(0.5, 0.4)	34	(0.2, 0.0)	0.30	0.29	-0.00	-0.01	6
(0.7, 0.4)	112	0.9	(0.5, 0.4)	11	(0.2, 0.0)	0.30	0.29	-0.00	-0.01	3
(0.7, 0.4)	112	0.5	(0.7, 0.2)	56	(0.0, 0.2)	0.30	0.32	-0.00	0.02	6
(0.7, 0.4)	112	0.7	(0.7, 0.2)	34	(0.0, 0.2)	0.30	0.31	-0.00	0.01	5
(0.7, 0.4)	112	0.9	(0.7, 0.2)	11	(0.0, 0.2)	0.30	0.31	-0.00	0.01	4
(0.7, 0.5)	248	0.5	(0.1, 0.2)	124	(0.6, 0.3)	0.20	0.19	-0.00	-0.01	-2
(0.7, 0.5)	248	0.7	(0.1, 0.2)	74	(0.6, 0.3)	0.20	0.19	-0.00	-0.01	-1
(0.7, 0.5)	248	0.9	(0.1, 0.2)	25	(0.6, 0.3)	0.20	0.19	-0.00	-0.01	1
(0.7, 0.5)	248	0.5	(0.1, 0.4)	124	(0.6, 0.1)	0.20	0.19	-0.00	-0.01	-1
(0.7, 0.5)	248	0.7	(0.1, 0.4)	74	(0.6, 0.1)	0.20	0.19	-0.00	-0.01	-1
(0.7, 0.5)	248	0.9	(0.1, 0.4)	25	(0.6, 0.1)	0.20	0.19	-0.00	-0.01	-0
(0.7, 0.5)	248	0.5	(0.3, 0.2)	124	(0.4, 0.3)	0.20	0.19	-0.00	-0.01	7
(0.7, 0.5)	248	0.7	(0.3, 0.2)	74	(0.4, 0.3)	0.20	0.19	-0.00	-0.01	6
(0.7, 0.5)	248	0.9	(0.3, 0.2)	25	(0.4, 0.3)	0.20	0.20	-0.00	-0.00	3
(0.7, 0.5)	248	0.5	(0.3, 0.4)	124	(0.4, 0.1)	0.20	0.19	-0.00	-0.01	-1
(0.7, 0.5)	248	0.7	(0.3, 0.4)	74	(0.4, 0.1)	0.20	0.19	-0.00	-0.01	0
(0.7, 0.5)	248	0.9	(0.3, 0.4)	25	(0.4, 0.1)	0.20	0.19	-0.00	-0.01	2
(0.7, 0.5)	248	0.5	(0.5, 0.2)	124	(0.2, 0.3)	0.20	0.21	-0.00	0.01	6
(0.7, 0.5)	248	0.7	(0.5, 0.2)	74	(0.2, 0.3)	0.20	0.21	-0.00	0.01	5
(0.7, 0.5)	248	0.9	(0.5, 0.2)	25	(0.2, 0.3)	0.20	0.20	-0.00	0.00	3
(0.7, 0.5)	248	0.5	(0.5, 0.4)	124	(0.2, 0.1)	0.20	0.19	-0.00	-0.01	7
(0.7, 0.5)	248	0.7	(0.5, 0.4)	74	(0.2, 0.1)	0.20	0.19	-0.00	-0.01	5
(0.7, 0.5)	248	0.9	(0.5, 0.4)	25	(0.2, 0.1)	0.20	0.20	-0.00	-0.00	3
(0.7, 0.5)	248	0.5	(0.7, 0.2)	124	(0.0, 0.3)	0.20	0.21	-0.00	0.01	-1
(0.7, 0.5)	248	0.7	(0.7, 0.2)	74	(0.0, 0.3)	0.20	0.21	-0.00	0.01	-0
(0.7, 0.5)	248	0.9	(0.7, 0.2)	25	(0.0, 0.3)	0.20	0.21	-0.00	0.01	1
(0.7, 0.5)	248	0.5	(0.7, 0.4)	124	(0.0, 0.1)	0.20	0.21	-0.00	0.01	6
(0.7, 0.5)	248	0.7	(0.7, 0.4)	74	(0.0, 0.1)	0.20	0.21	-0.00	0.01	5
(0.7, 0.5)	248	0.9	(0.7, 0.4)	25	(0.0, 0.1)	0.20	0.20	-0.00	0.00	3

Table 5: Complement to meta-analysis results for CP, when $\pi_I^{\text{trial}} = 0.8$ and $\pi_C^{\text{trial}} = 0.5$, over 5,000 replications. Mean difference between the observed response rates at the end of the trial (d^{trial}) and corresponding bias ($B(d^{\text{trial}})$), mean estimated difference between the response rates after meta-analysis ($\hat{\Delta}^{\text{trial}}$) and corresponding bias $B(\hat{\Delta}^{\text{trial}})$, and mean proportion of gain (%) in effective sample size when using the shrinkage estimate relative to the original "plain" CI (gain). The gain in effective sample size is approximated as $(q^{\text{trial}})^{-2} - 1$ where $q^{\text{trial}} = \delta^{\text{trial}}/(2 \times 1.96\sigma^{\text{trial}})$ is the *relative width* and δ^{trial} the 95% shrinkage interval width.
CP: conditional power; CI: confidence interval; π_I^{trial}, π_C^{trial}: true response rates associated to intervention and control arms for the clinical trial population; n: total number of individuals in the trial; t: information time; π_I^{RWD}, π_C^{RWD}: true response rates associated to intervention and control arms for the RWD population; m: total sample size of the RWD population; $\text{drift}_I = \pi_I^{\text{trial}} - \pi_I^{\text{RWD}}$: drift between the clinical trial and RWD true responses rates associated to intervention arm; $\text{drift}_C = \pi_C^{\text{trial}} - \pi_C^{\text{RWD}}$: drift between the clinical trial and RWD true responses rates associated to control arm.

$(\pi_I^{\text{trial}}, \pi_C^{\text{trial}})$	**n**	**t**	$(\pi_I^{\text{RWD}}, \pi_C^{\text{RWD}})$	**m**	$(\text{drift}_I, \text{drift}_C)$	$\mathbf{d}^{\text{trial}}$	$\hat{\boldsymbol{\Delta}}^{\text{trial}}$	$\mathbf{B}(\mathbf{d}^{\text{trial}})$	$\mathbf{B}(\hat{\boldsymbol{\Delta}}^{\text{trial}})$	**gain (%)**
(0.8, 0.5)	104	0.5	(0.2, 0.2)	52	(0.6, 0.3)	0.30	0.28	-0.00	-0.02	2
(0.8, 0.5)	104	0.7	(0.2, 0.2)	31	(0.6, 0.3)	0.30	0.28	-0.00	-0.02	3
(0.8, 0.5)	104	0.9	(0.2, 0.2)	10	(0.6, 0.3)	0.30	0.29	-0.00	-0.01	3
(0.8, 0.5)	104	0.5	(0.2, 0.4)	52	(0.6, 0.1)	0.30	0.28	-0.00	-0.02	-1
(0.8, 0.5)	104	0.7	(0.2, 0.4)	31	(0.6, 0.1)	0.30	0.28	-0.00	-0.02	-0
(0.8, 0.5)	104	0.9	(0.2, 0.4)	10	(0.6, 0.1)	0.30	0.28	-0.00	-0.02	2
(0.8, 0.5)	104	0.5	(0.4, 0.2)	52	(0.4, 0.3)	0.30	0.29	-0.00	-0.01	10
(0.8, 0.5)	104	0.7	(0.4, 0.2)	31	(0.4, 0.3)	0.30	0.29	-0.00	-0.01	7
(0.8, 0.5)	104	0.9	(0.4, 0.2)	10	(0.4, 0.3)	0.30	0.29	-0.00	-0.01	4
(0.8, 0.5)	104	0.5	(0.4, 0.4)	52	(0.4, 0.1)	0.30	0.28	-0.00	-0.02	3
(0.8, 0.5)	104	0.7	(0.4, 0.4)	31	(0.4, 0.1)	0.30	0.28	-0.00	-0.02	3
(0.8, 0.5)	104	0.9	(0.4, 0.4)	10	(0.4, 0.1)	0.30	0.29	-0.00	-0.01	3
(0.8, 0.5)	104	0.5	(0.6, 0.2)	52	(0.2, 0.3)	0.30	0.31	-0.00	0.01	9
(0.8, 0.5)	104	0.7	(0.6, 0.2)	31	(0.2, 0.3)	0.30	0.31	-0.00	0.01	7
(0.8, 0.5)	104	0.9	(0.6, 0.2)	10	(0.2, 0.3)	0.30	0.30	-0.00	0.00	4
(0.8, 0.5)	104	0.5	(0.6, 0.4)	52	(0.2, 0.1)	0.30	0.29	-0.00	-0.01	9
(0.8, 0.5)	104	0.7	(0.6, 0.4)	31	(0.2, 0.1)	0.30	0.29	-0.00	-0.01	7
(0.8, 0.5)	104	0.9	(0.6, 0.4)	10	(0.2, 0.1)	0.30	0.30	-0.00	-0.00	3
(0.8, 0.5)	104	0.5	(0.8, 0.2)	52	(0.0, 0.3)	0.30	0.32	-0.00	0.02	2
(0.8, 0.5)	104	0.7	(0.8, 0.2)	31	(0.0, 0.3)	0.30	0.32	-0.00	0.02	2
(0.8, 0.5)	104	0.9	(0.8, 0.2)	10	(0.0, 0.3)	0.30	0.31	-0.00	0.01	3
(0.8, 0.5)	104	0.5	(0.8, 0.4)	52	(0.0, 0.1)	0.30	0.31	-0.00	0.01	9
(0.8, 0.5)	104	0.7	(0.8, 0.4)	31	(0.0, 0.1)	0.30	0.31	-0.00	0.01	7
(0.8, 0.5)	104	0.9	(0.8, 0.4)	10	(0.0, 0.1)	0.30	0.30	-0.00	0.00	4

Table 6: Complement to meta-analysis results for CP, when $\pi_I^{\text{trial}} = 0.8$ and $\pi_C^{\text{trial}} = 0.6$, over 5,000 replications. Mean difference between the observed response rates at the end of the trial (d^{trial}) and corresponding bias ($B(d^{\text{trial}})$), mean estimated difference between the response rates after meta-analysis ($\hat{\Delta}^{\text{trial}}$) and corresponding bias $B(\hat{\Delta}^{\text{trial}})$, and mean proportion of gain (%) in effective sample size when using the shrinkage estimate relative to the original "plain" CI (gain). The gain in effective sample size is approximated as $(q^{\text{trial}})^{-2} - 1$ where $q^{\text{trial}} = \delta^{\text{trial}}/(2 \times 1.96\sigma^{\text{trial}})$ is the *relative width* and δ^{trial} the 95% shrinkage interval width.
CP: conditional power; CI: confidence interval; π_I^{trial}, π_C^{trial}: true response rates associated to intervention and control arms for the clinical trial population; n: total number of individuals in the trial; t: information time; π_I^{RWD}, π_C^{RWD}: true response rates associated to intervention and control arms for the RWD population; m: total sample size of the RWD population; $\text{drift}_I = \pi_I^{\text{trial}} - \pi_I^{\text{RWD}}$: drift between the clinical trial and RWD true responses rates associated to intervention arm; $\text{drift}_C = \pi_C^{\text{trial}} - \pi_C^{\text{RWD}}$: drift between the clinical trial and RWD true responses rates associated to control arm.

$(\pi_I^{\text{trial}}, \pi_C^{\text{trial}})$	n	t	$(\pi_I^{\text{RWD}}, \pi_C^{\text{RWD}})$	m	$(\text{drift}_I, \text{drift}_C)$	d^{trial}	$\hat{\Delta}^{\text{trial}}$	$B(d^{\text{trial}})$	$B(\hat{\Delta}^{\text{trial}})$	gain (%)
(0.8, 0.6)	218	0.5	(0.2, 0.2)	109	(0.6, 0.4)	0.20	0.19	-0.00	-0.01	2
(0.8, 0.6)	218	0.7	(0.2, 0.2)	65	(0.6, 0.4)	0.20	0.19	-0.00	-0.01	2
(0.8, 0.6)	218	0.9	(0.2, 0.2)	22	(0.6, 0.4)	0.20	0.19	-0.00	-0.01	2
(0.8, 0.6)	218	0.5	(0.2, 0.4)	109	(0.6, 0.2)	0.20	0.19	-0.00	-0.01	-1
(0.8, 0.6)	218	0.7	(0.2, 0.4)	65	(0.6, 0.2)	0.20	0.19	-0.00	-0.01	-1
(0.8, 0.6)	218	0.9	(0.2, 0.4)	22	(0.6, 0.2)	0.20	0.19	-0.00	-0.01	1
(0.8, 0.6)	218	0.5	(0.4, 0.2)	109	(0.4, 0.4)	0.20	0.20	-0.00	-0.00	9
(0.8, 0.6)	218	0.7	(0.4, 0.2)	65	(0.4, 0.4)	0.20	0.20	-0.00	-0.00	6
(0.8, 0.6)	218	0.9	(0.4, 0.2)	22	(0.4, 0.4)	0.20	0.20	-0.00	-0.00	3
(0.8, 0.6)	218	0.5	(0.4, 0.4)	109	(0.4, 0.2)	0.20	0.19	-0.00	-0.01	2
(0.8, 0.6)	218	0.7	(0.4, 0.4)	65	(0.4, 0.2)	0.20	0.19	-0.00	-0.01	3
(0.8, 0.6)	218	0.9	(0.4, 0.4)	22	(0.4, 0.2)	0.20	0.19	-0.00	-0.01	2
(0.8, 0.6)	218	0.5	(0.4, 0.6)	109	(0.4, 0.0)	0.20	0.19	-0.00	-0.01	-1
(0.8, 0.6)	218	0.7	(0.4, 0.6)	65	(0.4, 0.0)	0.20	0.19	-0.00	-0.01	-0
(0.8, 0.6)	218	0.9	(0.4, 0.6)	22	(0.4, 0.0)	0.20	0.19	-0.00	-0.01	1
(0.8, 0.6)	218	0.5	(0.6, 0.2)	109	(0.2, 0.4)	0.20	0.21	-0.00	0.01	2
(0.8, 0.6)	218	0.7	(0.6, 0.2)	65	(0.2, 0.4)	0.20	0.21	-0.00	0.01	2
(0.8, 0.6)	218	0.9	(0.6, 0.2)	22	(0.2, 0.4)	0.20	0.20	-0.00	0.00	2
(0.8, 0.6)	218	0.5	(0.6, 0.4)	109	(0.2, 0.2)	0.20	0.20	-0.00	-0.00	8
(0.8, 0.6)	218	0.7	(0.6, 0.4)	65	(0.2, 0.2)	0.20	0.20	-0.00	-0.00	6
(0.8, 0.6)	218	0.9	(0.6, 0.4)	22	(0.2, 0.2)	0.20	0.20	-0.00	-0.00	3
(0.8, 0.6)	218	0.5	(0.6, 0.6)	109	(0.2, 0.0)	0.20	0.19	-0.00	-0.01	2
(0.8, 0.6)	218	0.7	(0.6, 0.6)	65	(0.2, 0.0)	0.20	0.19	-0.00	-0.01	3
(0.8, 0.6)	218	0.9	(0.6, 0.6)	22	(0.2, 0.0)	0.20	0.19	-0.00	-0.01	2
(0.8, 0.6)	218	0.5	(0.8, 0.2)	109	(0.0, 0.4)	0.20	0.21	-0.00	0.01	-1
(0.8, 0.6)	218	0.7	(0.8, 0.2)	65	(0.0, 0.4)	0.20	0.21	-0.00	0.01	-1
(0.8, 0.6)	218	0.9	(0.8, 0.2)	22	(0.0, 0.4)	0.20	0.21	-0.00	0.01	0
(0.8, 0.6)	218	0.5	(0.8, 0.4)	109	(0.0, 0.2)	0.20	0.21	-0.00	0.01	2
(0.8, 0.6)	218	0.7	(0.8, 0.4)	65	(0.0, 0.2)	0.20	0.21	-0.00	0.01	2
(0.8, 0.6)	218	0.9	(0.8, 0.4)	22	(0.0, 0.2)	0.20	0.20	-0.00	0.00	2

Table 7: Complement to meta-analysis results for CP, when $\pi_I^{\text{trial}} = 0.9$ and $\pi_C^{\text{trial}} = 0.6$, over 5,000 replications. Mean difference between the observed response rates at the end of the trial (d^{trial}) and corresponding bias ($B(d^{\text{trial}})$), mean estimated difference between the response rates after meta-analysis ($\hat{\Delta}^{\text{trial}}$) and corresponding bias $B(\hat{\Delta}^{\text{trial}})$, and mean proportion of gain (%) in effective sample size when using the shrinkage estimate relative to the original "plain" CI (gain). The gain in effective sample size is approximated as $(q^{\text{trial}})^{-2} - 1$ where $q^{\text{trial}} = \delta^{\text{trial}}/(2 \times 1.96\sigma^{\text{trial}})$ is the *relative width* and δ^{trial} the 95% shrinkage interval width.

CP: conditional power; CI: confidence interval; π_I^{trial}, π_C^{trial}: true response rates associated to intervention and control arms for the clinical trial population; n: total number of individuals in the trial; t: information time; π_I^{RWD}, π_C^{RWD}: true response rates associated to intervention and control arms for the RWD population; m: total sample size of the RWD population; $\text{drift}_I = \pi_I^{\text{trial}} - \pi_I^{\text{RWD}}$: drift between the clinical trial and RWD true responses rates associated to intervention arm; $\text{drift}_C = \pi_C^{\text{trial}} - \pi_C^{\text{RWD}}$: drift between the clinical trial and RWD true responses rates associated to control arm.

$(\pi_{\mathbf{I}}^{\text{trial}}, \pi_{\mathbf{C}}^{\text{trial}})$	**n**	**t**	$(\pi_{\mathbf{I}}^{\text{RWD}}, \pi_{\mathbf{C}}^{\text{RWD}})$	**m**	$(\text{drift}_I, \text{drift}_C)$	$\mathbf{d}^{\text{trial}}$	$\hat{\mathbf{\Delta}}^{\text{trial}}$	$\mathbf{B}(\mathbf{d}^{\text{trial}})$	$\mathbf{B}(\hat{\mathbf{\Delta}}^{\text{trial}})$	**gain (%)**
(0.9, 0.6)	84	0.5	(0.1, 0.2)	42	(0.8, 0.4)	0.30	0.28	0.00	-0.02	-1
(0.9, 0.6)	84	0.7	(0.1, 0.2)	25	(0.8, 0.4)	0.30	0.28	0.00	-0.02	0
(0.9, 0.6)	84	0.9	(0.1, 0.2)	8	(0.8, 0.4)	0.30	0.29	0.00	-0.01	2
(0.9, 0.6)	84	0.5	(0.1, 0.4)	42	(0.8, 0.2)	0.30	0.28	0.00	-0.02	-1
(0.9, 0.6)	84	0.7	(0.1, 0.4)	25	(0.8, 0.2)	0.30	0.28	0.00	-0.02	-1
(0.9, 0.6)	84	0.9	(0.1, 0.4)	8	(0.8, 0.2)	0.30	0.29	0.00	-0.01	1
(0.9, 0.6)	84	0.5	(0.1, 0.6)	42	(0.8, 0.0)	0.30	0.29	0.00	-0.01	-1
(0.9, 0.6)	84	0.7	(0.1, 0.6)	25	(0.8, 0.0)	0.30	0.29	0.00	-0.01	-1
(0.9, 0.6)	84	0.9	(0.1, 0.6)	8	(0.8, 0.0)	0.30	0.29	0.00	-0.01	0
(0.9, 0.6)	84	0.5	(0.3, 0.2)	42	(0.6, 0.4)	0.30	0.29	0.00	-0.01	6
(0.9, 0.6)	84	0.7	(0.3, 0.2)	25	(0.6, 0.4)	0.30	0.29	0.00	-0.01	5
(0.9, 0.6)	84	0.9	(0.3, 0.2)	8	(0.6, 0.4)	0.30	0.29	0.00	-0.01	3
(0.9, 0.6)	84	0.5	(0.3, 0.4)	42	(0.6, 0.2)	0.30	0.28	0.00	-0.02	1
(0.9, 0.6)	84	0.7	(0.3, 0.4)	25	(0.6, 0.2)	0.30	0.29	0.00	-0.01	2
(0.9, 0.6)	84	0.9	(0.3, 0.4)	8	(0.6, 0.2)	0.30	0.29	0.00	-0.01	2
(0.9, 0.6)	84	0.5	(0.5, 0.2)	42	(0.4, 0.4)	0.30	0.30	0.00	0.00	9
(0.9, 0.6)	84	0.7	(0.5, 0.2)	25	(0.4, 0.4)	0.30	0.30	0.00	0.00	7
(0.9, 0.6)	84	0.9	(0.5, 0.2)	8	(0.4, 0.4)	0.30	0.30	0.00	0.00	4
(0.9, 0.6)	84	0.5	(0.5, 0.4)	42	(0.4, 0.2)	0.30	0.29	0.00	-0.01	6
(0.9, 0.6)	84	0.7	(0.5, 0.4)	25	(0.4, 0.2)	0.30	0.29	0.00	-0.01	5
(0.9, 0.6)	84	0.9	(0.5, 0.4)	8	(0.4, 0.2)	0.30	0.30	0.00	-0.00	3
(0.9, 0.6)	84	0.5	(0.5, 0.6)	42	(0.4, 0.0)	0.30	0.28	0.00	-0.02	1
(0.9, 0.6)	84	0.7	(0.5, 0.6)	25	(0.4, 0.0)	0.30	0.29	0.00	-0.01	2
(0.9, 0.6)	84	0.9	(0.5, 0.6)	8	(0.4, 0.0)	0.30	0.29	0.00	-0.01	2
(0.9, 0.6)	84	0.5	(0.7, 0.2)	42	(0.2, 0.4)	0.30	0.32	0.00	0.02	5
(0.9, 0.6)	84	0.7	(0.7, 0.2)	25	(0.2, 0.4)	0.30	0.31	0.00	0.01	5
(0.9, 0.6)	84	0.9	(0.7, 0.2)	8	(0.2, 0.4)	0.30	0.31	0.00	0.01	3
(0.9, 0.6)	84	0.5	(0.7, 0.4)	42	(0.2, 0.2)	0.30	0.30	0.00	0.00	9
(0.9, 0.6)	84	0.7	(0.7, 0.4)	25	(0.2, 0.2)	0.30	0.30	0.00	0.00	6
(0.9, 0.6)	84	0.9	(0.7, 0.4)	8	(0.2, 0.2)	0.30	0.30	0.00	0.00	3
(0.9, 0.6)	84	0.5	(0.7, 0.6)	42	(0.2, 0.0)	0.30	0.29	0.00	-0.01	6
(0.9, 0.6)	84	0.7	(0.7, 0.6)	25	(0.2, 0.0)	0.30	0.29	0.00	-0.01	5
(0.9, 0.6)	84	0.9	(0.7, 0.6)	8	(0.2, 0.0)	0.30	0.30	0.00	-0.00	3
(0.9, 0.6)	84	0.5	(0.9, 0.2)	42	(0.0, 0.4)	0.30	0.32	0.00	0.02	-1
(0.9, 0.6)	84	0.7	(0.9, 0.2)	25	(0.0, 0.4)	0.30	0.32	0.00	0.02	1
(0.9, 0.6)	84	0.9	(0.9, 0.2)	8	(0.0, 0.4)	0.30	0.31	0.00	0.01	3
(0.9, 0.6)	84	0.5	(0.9, 0.4)	42	(0.0, 0.2)	0.30	0.32	0.00	0.02	6
(0.9, 0.6)	84	0.7	(0.9, 0.4)	25	(0.0, 0.2)	0.30	0.31	0.00	0.01	5
(0.9, 0.6)	84	0.9	(0.9, 0.4)	8	(0.0, 0.2)	0.30	0.31	0.00	0.01	4

Table 8: Complement to meta-analysis results for CP, when $\pi_I^{\text{trial}} = 0.9$ and $\pi_C^{\text{trial}} = 0.7$, over 5,000 replications. Mean difference between the observed response rates at the end of the trial (d^{trial}) and corresponding bias ($B(d^{\text{trial}})$), mean estimated difference between the response rates after meta-analysis ($\hat{\Delta}^{\text{trial}}$) and corresponding bias $B(\hat{\Delta}^{\text{trial}})$, and mean proportion of gain (%) in effective sample size when using the shrinkage estimate relative to the original "plain" CI (gain). The gain in effective sample size is approximated as $(q^{\text{trial}})^{-2} - 1$ where $q^{\text{trial}} = \delta^{\text{trial}}/(2 \times 1.96\sigma^{\text{trial}})$ is the *relative width* and δ^{trial} the 95% shrinkage interval width.
CP: conditional power; CI: confidence interval; π_I^{trial}, π_C^{trial}: true response rates associated to intervention and control arms for the clinical trial population; n: total number of individuals in the trial; t: information time; π_I^{RWD}, π_C^{RWD}: true response rates associated to intervention and control arms for the RWD population; m: total sample size of the RWD population; $\text{drift}_I = \pi_I^{\text{trial}} - \pi_I^{\text{RWD}}$: drift between the clinical trial and RWD true responses rates associated to intervention arm; $\text{drift}_C = \pi_C^{\text{trial}} - \pi_C^{\text{RWD}}$: drift between the clinical trial and RWD true responses rates associated to control arm.

$(\pi_I^{\text{trial}}, \pi_C^{\text{trial}})$	n	t	$(\pi_I^{\text{RWD}}, \pi_C^{\text{RWD}})$	m	$(\text{drift}_I, \text{drift}_C)$	d^{trial}	$\hat{\Delta}^{\text{trial}}$	$B(d^{\text{trial}})$	$B(\hat{\Delta}^{\text{trial}})$	gain (%)
(0.9, 0.7)	164	0.5	(0.1, 0.1)	82	(0.8, 0.6)	0.20	0.19	-0.00	-0.01	1
(0.9, 0.7)	164	0.7	(0.1, 0.1)	49	(0.8, 0.6)	0.20	0.19	-0.00	-0.01	2
(0.9, 0.7)	164	0.9	(0.1, 0.1)	16	(0.8, 0.6)	0.20	0.19	-0.00	-0.01	2
(0.9, 0.7)	164	0.5	(0.1, 0.3)	82	(0.8, 0.4)	0.20	0.19	-0.00	-0.01	-1
(0.9, 0.7)	164	0.7	(0.1, 0.3)	49	(0.8, 0.4)	0.20	0.19	-0.00	-0.01	-1
(0.9, 0.7)	164	0.9	(0.1, 0.3)	16	(0.8, 0.4)	0.20	0.19	-0.00	-0.01	1
(0.9, 0.7)	164	0.5	(0.1, 0.5)	82	(0.8, 0.2)	0.20	0.19	-0.00	-0.01	-1
(0.9, 0.7)	164	0.7	(0.1, 0.5)	49	(0.8, 0.2)	0.20	0.19	-0.00	-0.01	-1
(0.9, 0.7)	164	0.9	(0.1, 0.5)	16	(0.8, 0.2)	0.20	0.19	-0.00	-0.01	-0
(0.9, 0.7)	164	0.5	(0.1, 0.7)	82	(0.8, 0.0)	0.20	0.19	-0.00	-0.01	-0
(0.9, 0.7)	164	0.7	(0.1, 0.7)	49	(0.8, 0.0)	0.20	0.19	-0.00	-0.01	-0
(0.9, 0.7)	164	0.9	(0.1, 0.7)	16	(0.8, 0.0)	0.20	0.19	-0.00	-0.01	-0
(0.9, 0.7)	164	0.5	(0.3, 0.1)	82	(0.6, 0.6)	0.20	0.20	-0.00	-0.00	9
(0.9, 0.7)	164	0.7	(0.3, 0.1)	49	(0.6, 0.6)	0.20	0.20	-0.00	-0.00	6
(0.9, 0.7)	164	0.9	(0.3, 0.1)	16	(0.6, 0.6)	0.20	0.20	-0.00	-0.00	3
(0.9, 0.7)	164	0.5	(0.3, 0.3)	82	(0.6, 0.4)	0.20	0.19	-0.00	-0.01	2
(0.9, 0.7)	164	0.7	(0.3, 0.3)	49	(0.6, 0.4)	0.20	0.19	-0.00	-0.01	3
(0.9, 0.7)	164	0.9	(0.3, 0.3)	16	(0.6, 0.4)	0.20	0.19	-0.00	-0.01	2
(0.9, 0.7)	164	0.5	(0.3, 0.5)	82	(0.6, 0.2)	0.20	0.19	-0.00	-0.01	-1
(0.9, 0.7)	164	0.7	(0.3, 0.5)	49	(0.6, 0.2)	0.20	0.19	-0.00	-0.01	-0
(0.9, 0.7)	164	0.9	(0.3, 0.5)	16	(0.6, 0.2)	0.20	0.19	-0.00	-0.01	1
(0.9, 0.7)	164	0.5	(0.5, 0.1)	82	(0.4, 0.6)	0.20	0.21	-0.00	0.01	2
(0.9, 0.7)	164	0.7	(0.5, 0.1)	49	(0.4, 0.6)	0.20	0.21	-0.00	0.01	2
(0.9, 0.7)	164	0.9	(0.5, 0.1)	16	(0.4, 0.6)	0.20	0.20	-0.00	0.00	2
(0.9, 0.7)	164	0.5	(0.5, 0.3)	82	(0.4, 0.4)	0.20	0.20	-0.00	-0.00	7
(0.9, 0.7)	164	0.7	(0.5, 0.3)	49	(0.4, 0.4)	0.20	0.20	-0.00	-0.00	5
(0.9, 0.7)	164	0.9	(0.5, 0.3)	16	(0.4, 0.4)	0.20	0.20	-0.00	-0.00	2
(0.9, 0.7)	164	0.5	(0.5, 0.5)	82	(0.4, 0.2)	0.20	0.19	-0.00	-0.01	3
(0.9, 0.7)	164	0.7	(0.5, 0.5)	49	(0.4, 0.2)	0.20	0.19	-0.00	-0.01	3
(0.9, 0.7)	164	0.9	(0.5, 0.5)	16	(0.4, 0.2)	0.20	0.20	-0.00	-0.00	2
(0.9, 0.7)	164	0.5	(0.5, 0.7)	82	(0.4, 0.0)	0.20	0.19	-0.00	-0.01	-1
(0.9, 0.7)	164	0.7	(0.5, 0.7)	49	(0.4, 0.0)	0.20	0.19	-0.00	-0.01	-0
(0.9, 0.7)	164	0.9	(0.5, 0.7)	16	(0.4, 0.0)	0.20	0.19	-0.00	-0.01	1
(0.9, 0.7)	164	0.5	(0.7, 0.1)	82	(0.2, 0.6)	0.20	0.21	-0.00	0.01	-1
(0.9, 0.7)	164	0.7	(0.7, 0.1)	49	(0.2, 0.6)	0.20	0.21	-0.00	0.01	-1
(0.9, 0.7)	164	0.9	(0.7, 0.1)	16	(0.2, 0.6)	0.20	0.21	-0.00	0.01	1
(0.9, 0.7)	164	0.5	(0.7, 0.3)	82	(0.2, 0.4)	0.20	0.21	-0.00	0.01	2
(0.9, 0.7)	164	0.7	(0.7, 0.3)	49	(0.2, 0.4)	0.20	0.21	-0.00	0.01	2
(0.9, 0.7)	164	0.9	(0.7, 0.3)	16	(0.2, 0.4)	0.20	0.20	-0.00	0.00	2
(0.9, 0.7)	164	0.5	(0.7, 0.5)	82	(0.2, 0.2)	0.20	0.20	-0.00	-0.00	7
(0.9, 0.7)	164	0.7	(0.7, 0.5)	49	(0.2, 0.2)	0.20	0.20	-0.00	-0.00	5
(0.9, 0.7)	164	0.9	(0.7, 0.5)	16	(0.2, 0.2)	0.20	0.20	-0.00	-0.00	2
(0.9, 0.7)	164	0.5	(0.7, 0.7)	82	(0.2, 0.0)	0.20	0.19	-0.00	-0.01	2
(0.9, 0.7)	164	0.7	(0.7, 0.7)	49	(0.2, 0.0)	0.20	0.19	-0.00	-0.01	3
(0.9, 0.7)	164	0.9	(0.7, 0.7)	16	(0.2, 0.0)	0.20	0.19	-0.00	-0.01	2
(0.9, 0.7)	164	0.5	(0.9, 0.1)	82	(0.0, 0.6)	0.20	0.21	-0.00	0.01	-1
(0.9, 0.7)	164	0.7	(0.9, 0.1)	49	(0.0, 0.6)	0.20	0.21	-0.00	0.01	-1
(0.9, 0.7)	164	0.9	(0.9, 0.1)	16	(0.0, 0.6)	0.20	0.21	-0.00	0.01	-0
(0.9, 0.7)	164	0.5	(0.9, 0.3)	82	(0.0, 0.4)	0.20	0.21	-0.00	0.01	-1
(0.9, 0.7)	164	0.7	(0.9, 0.3)	49	(0.0, 0.4)	0.20	0.21	-0.00	0.01	-1
(0.9, 0.7)	164	0.9	(0.9, 0.3)	16	(0.0, 0.4)	0.20	0.21	-0.00	0.01	1
(0.9, 0.7)	164	0.5	(0.9, 0.5)	82	(0.0, 0.2)	0.20	0.21	-0.00	0.01	2
(0.9, 0.7)	164	0.7	(0.9, 0.5)	49	(0.0, 0.2)	0.20	0.21	-0.00	0.01	2
(0.9, 0.7)	164	0.9	(0.9, 0.5)	16	(0.0, 0.2)	0.20	0.20	-0.00	0.00	2

Table 9: Complement to interim and final clinical trial results for CP and $t \in \{0.1, 0.2, 0.3, 0.4, 0.5, 0.6\}$ over 5,000 replications. Mean and standard deviation of CP value, frequency (on all the 5,000 replications) when the p-value is lower than $\alpha = 0.025$ for the final analysis, frequencies when the CP value is greater than 0.6, 0.7 and 0.8 and frequencies (only for the corresponding replications) when the p-value is lower than $\alpha = 0.025$ for the final analysis. CP: conditional power; π_I^{trial}, π_C^{trial}: true response rates associated to intervention and control arms for the clinical trial population; n: total number of individuals in the trial; t: information time; pval: p-value for the final analysis.

						**CP ≥ 0.6**		**CP ≥ 0.7**		**CP ≥ 0.8**	
π_I^{trial}	π_C^{trial}	**n**	**t**	**CP**	**pval ≤ α**	**Yes**	**pval ≤ α**	**Yes**	**pval ≤ α**	**Yes**	**pval ≤ α**
0.7	0.4	112	0.1	0.67 (0.41)	0.89	0.65	0.92	0.53	0.93	0.53	0.93
0.7	0.4	112	0.2	0.72 (0.37)	0.89	0.66	0.94	0.65	0.94	0.65	0.94
0.7	0.4	112	0.5	0.80 (0.30)	0.89	0.79	0.96	0.75	0.96	0.70	0.97
0.7	0.5	248	0.1	0.66 (0.41)	0.90	0.65	0.93	0.65	0.93	0.57	0.93
0.7	0.5	248	0.2	0.71 (0.37)	0.90	0.66	0.94	0.66	0.94	0.60	0.94
0.7	0.5	248	0.5	0.80 (0.30)	0.90	0.79	0.96	0.76	0.97	0.71	0.97
0.8	0.5	104	0.1	0.69 (0.40)	0.91	0.71	0.94	0.57	0.95	0.57	0.95
0.8	0.5	104	0.2	0.73 (0.36)	0.91	0.71	0.95	0.65	0.95	0.61	0.96
0.8	0.5	104	0.4	0.78 (0.32)	0.91	0.78	0.96	0.72	0.97	0.71	0.97
0.8	0.6	218	0.1	0.66 (0.41)	0.90	0.61	0.94	0.61	0.94	0.61	0.94
0.8	0.6	218	0.2	0.71 (0.37)	0.90	0.71	0.95	0.63	0.96	0.61	0.96
0.8	0.6	218	0.5	0.79 (0.30)	0.90	0.78	0.96	0.75	0.97	0.69	0.98
0.9	0.6	84	0.1	0.77 (0.38)	0.92	0.81	0.94	0.68	0.94	0.68	0.94
0.9	0.6	84	0.2	0.75 (0.36)	0.92	0.76	0.95	0.76	0.95	0.69	0.95
0.9	0.6	84	0.4	0.80 (0.30)	0.92	0.76	0.96	0.76	0.96	0.69	0.97
0.9	0.7	164	0.1	0.71 (0.40)	0.90	0.63	0.94	0.63	0.94	0.63	0.94
0.9	0.7	164	0.2	0.73 (0.36)	0.90	0.70	0.95	0.69	0.96	0.64	0.96
0.9	0.7	164	0.5	0.81 (0.30)	0.90	0.81	0.97	0.75	0.98	0.70	0.98

Table 10: Complement to meta-analysis results for CP and $t \in \{0.1, 0.2, 0.3, 0.4, 0.5, 0.6\}$ over 5,000 replications. Mean difference between the observed response rates at the end of the trial (d^{trial}) and corresponding bias ($B(d^{\text{trial}})$), mean estimated difference between the response rates after meta-analysis ($\hat{\Delta}^{\text{trial}}$) and corresponding bias $B(\hat{\Delta}^{\text{trial}})$, and mean proportion of gain (%) in effective sample size when using the shrinkage estimate relative to the original "plain" CI (gain). The gain in effective sample size is approximated as $(q^{\text{trial}})^{-2} - 1$ where $q^{\text{trial}} = \delta^{\text{trial}}/(2 \times 1.96\sigma^{\text{trial}})$ is the *relative width* and δ^{trial} the 95% shrinkage interval width.
CP: conditional power; CI: confidence interval; π_I^{trial}, π_C^{trial}: true response rates associated to intervention and control arms for the clinical trial population; n: total number of individuals in the trial; t: information time; π_I^{RWD}, π_C^{RWD}: true response rates associated to intervention and control arms for the RWD population; m: total sample size of the RWD population.

$\pi_{\mathbf{I}}^{\text{trial}}$	$\pi_{\mathbf{C}}^{\text{trial}}$	**n**	**t**	$\pi_{\mathbf{I}}^{\text{RWD}}$	$\pi_{\mathbf{C}}^{\text{RWD}}$	**m**	$\mathbf{d}^{\text{trial}}$	$\hat{\mathbf{\Delta}}^{\text{trial}}$	$\mathbf{B}(\mathbf{d}^{\text{trial}})$	$\mathbf{B}(\hat{\mathbf{\Delta}}^{\text{trial}})$	**gain (%)**
0.7	0.4	112	0.1	0.1	0.4	101	0.30	0.28	-0.00	-0.02	-1
0.7	0.4	112	0.2	0.1	0.4	90	0.30	0.28	-0.00	-0.02	-1
0.7	0.4	112	0.5	0.1	0.4	56	0.30	0.28	-0.00	-0.02	-1
0.7	0.4	112	0.1	0.7	0.4	101	0.30	0.30	-0.00	-0.00	14
0.7	0.4	112	0.2	0.7	0.4	90	0.30	0.30	-0.00	-0.00	14
0.7	0.4	112	0.5	0.7	0.4	56	0.30	0.30	-0.00	-0.00	11
0.7	0.5	248	0.1	0.1	0.5	223	0.20	0.19	-0.00	-0.01	0
0.7	0.5	248	0.2	0.1	0.5	198	0.20	0.19	-0.00	-0.01	0
0.7	0.5	248	0.5	0.1	0.5	124	0.20	0.19	-0.00	-0.01	-1
0.7	0.5	248	0.1	0.7	0.5	223	0.20	0.20	-0.00	-0.00	12
0.7	0.5	248	0.2	0.7	0.5	198	0.20	0.20	-0.00	-0.00	11
0.7	0.5	248	0.5	0.7	0.5	124	0.20	0.20	-0.00	-0.00	9
0.8	0.5	104	0.1	0.2	0.5	94	0.30	0.28	-0.00	-0.02	-1
0.8	0.5	104	0.2	0.2	0.5	83	0.30	0.28	-0.00	-0.02	-1
0.8	0.5	104	0.4	0.2	0.5	62	0.30	0.28	-0.00	-0.02	-1
0.8	0.5	104	0.1	0.8	0.5	94	0.30	0.30	-0.00	-0.00	14
0.8	0.5	104	0.2	0.8	0.5	83	0.30	0.30	-0.00	-0.00	14
0.8	0.5	104	0.4	0.8	0.5	62	0.30	0.30	-0.00	-0.00	12
0.8	0.6	218	0.1	0.2	0.6	196	0.20	0.19	-0.00	-0.01	-1
0.8	0.6	218	0.2	0.2	0.6	174	0.20	0.19	-0.00	-0.01	-1
0.8	0.6	218	0.5	0.2	0.6	109	0.20	0.19	-0.00	-0.01	-1
0.8	0.6	218	0.1	0.8	0.6	196	0.20	0.20	-0.00	-0.00	12
0.8	0.6	218	0.2	0.8	0.6	174	0.20	0.20	-0.00	-0.00	11
0.8	0.6	218	0.5	0.8	0.6	109	0.20	0.20	-0.00	-0.00	9
0.9	0.6	84	0.1	0.3	0.6	76	0.30	0.28	0.00	-0.02	-1
0.9	0.6	84	0.2	0.3	0.6	67	0.30	0.28	0.00	-0.02	-1
0.9	0.6	84	0.4	0.3	0.6	50	0.30	0.28	0.00	-0.02	-1
0.9	0.6	84	0.1	0.9	0.6	76	0.30	0.30	0.00	0.00	14
0.9	0.6	84	0.2	0.9	0.6	67	0.30	0.30	0.00	0.00	13
0.9	0.6	84	0.4	0.9	0.6	50	0.30	0.30	0.00	0.00	12
0.9	0.7	164	0.1	0.3	0.7	148	0.20	0.19	-0.00	-0.01	-1
0.9	0.7	164	0.2	0.3	0.7	131	0.20	0.19	-0.00	-0.01	-1
0.9	0.7	164	0.5	0.3	0.7	82	0.20	0.19	-0.00	-0.01	-1
0.9	0.7	164	0.1	0.9	0.7	148	0.20	0.20	-0.00	-0.00	12
0.9	0.7	164	0.2	0.9	0.7	131	0.20	0.20	-0.00	-0.00	11
0.9	0.7	164	0.5	0.9	0.7	82	0.20	0.20	-0.00	-0.00	9

Table 11: Complement to interim and final clinical trial results for PP and $t \in \{0.1, 0.2, 0.3, 0.4, 0.5, 0.6\}$ over 5,000 replications. Mean and standard deviation of PP value, frequency (on all the 5,000 replications) when the p-value is lower than $\alpha = 0.025$ for the final analysis, frequencies when the PP value is greater than 0.8, 0.85 and 0.9 and (only for the corresponding replications) when the p-value is lower than $\alpha = 0.025$ for the final analysis. PP: predictive power; π_I^{trial}, π_C^{trial}: true response rates associated to intervention and control arms for the clinical trial population; n: total number of individuals in the trial; t: information time; pval: p-value for the final analysis.

						**PP $\geq$ 0.8**		**PP $\geq$ 0.85**		**PP $\geq$ 0.9**	
$\pi_{\mathbf{I}}^{\text{trial}}$	$\pi_{\mathbf{C}}^{\text{trial}}$	**n**	**t**	**PP**	**pval $\leq \alpha$**	**Yes**	**pval $\leq \alpha$**	**Yes**	**pval $\leq \alpha$**	**Yes**	**pval $\leq \alpha$**
0.7	0.4	112	0.1	0.65 (0.31)	0.89	0.53	0.93	0.53	0.93	0.52	0.93
0.7	0.4	112	0.3	0.72 (0.29)	0.89	0.68	0.95	0.60	0.96	0.55	0.97
0.7	0.4	112	0.5	0.78 (0.28)	0.89	0.79	0.96	0.70	0.97	0.61	0.98
0.7	0.5	248	0.1	0.63 (0.30)	0.90	0.57	0.93	0.50	0.93	0.34	0.94
0.7	0.5	248	0.3	0.72 (0.28)	0.90	0.69	0.95	0.60	0.96	0.50	0.97
0.7	0.5	248	0.6	0.81 (0.27)	0.90	0.81	0.97	0.76	0.98	0.69	0.98
0.8	0.5	104	0.1	0.66 (0.30)	0.91	0.57	0.95	0.57	0.95	0.51	0.95
0.8	0.5	104	0.2	0.70 (0.29)	0.91	0.65	0.95	0.61	0.96	0.48	0.97
0.8	0.5	104	0.5	0.79 (0.27)	0.91	0.78	0.97	0.74	0.97	0.66	0.98
0.8	0.6	218	0.1	0.63 (0.30)	0.90	0.61	0.94	0.48	0.95	0.42	0.96
0.8	0.6	218	0.3	0.71 (0.29)	0.90	0.70	0.96	0.60	0.97	0.52	0.97
0.8	0.6	218	0.6	0.80 (0.27)	0.90	0.81	0.97	0.75	0.98	0.69	0.98
0.9	0.6	84	0.1	0.70 (0.29)	0.92	0.68	0.94	0.68	0.94	0.44	0.96
0.9	0.6	84	0.2	0.71 (0.28)	0.92	0.69	0.95	0.58	0.96	0.58	0.96
0.9	0.6	84	0.5	0.81 (0.26)	0.92	0.81	0.97	0.76	0.98	0.66	0.98
0.9	0.7	164	0.1	0.66 (0.29)	0.90	0.63	0.94	0.52	0.95	0.42	0.95
0.9	0.7	164	0.3	0.72 (0.28)	0.90	0.71	0.96	0.63	0.97	0.56	0.97
0.9	0.7	164	0.6	0.81 (0.27)	0.90	0.81	0.98	0.77	0.98	0.69	0.99

Table 12: Complement to meta-analysis results for PP and $t \in \{0.1, 0.2, 0.3, 0.4, 0.5, 0.6\}$ over 5,000 replications. Mean difference between the observed response rates at the end of the trial (d^{trial}) and corresponding bias ($B(d^{\text{trial}})$), mean estimated difference between the response rates after meta-analysis ($\hat{\Delta}^{\text{trial}}$) and corresponding bias $B(\hat{\Delta}^{\text{trial}})$, and mean proportion of gain (%) in effective sample size when using the shrinkage estimate relative to the original "plain" CI (gain). The gain in effective sample size is approximated as $(q^{\text{trial}})^{-2} - 1$ where $q^{\text{trial}} = \delta^{\text{trial}}/(2 \times 1.96\sigma^{\text{trial}})$ is the *relative width* and δ^{trial} the 95% shrinkage interval width.

PP: predictive power; CI: confidence interval; π_I^{trial}, π_C^{trial}: true response rates associated to intervention and control arms for the clinical trial population; n: total number of individuals in the trial; t: information time; π_I^{RWD}, π_C^{RWD}: true response rates associated to intervention and control arms for the RWD population; m: total sample size of the RWD population.

$\pi_{\mathbf{I}}^{\text{trial}}$	$\pi_{\mathbf{C}}^{\text{trial}}$	**n**	**t**	$\pi_{\mathbf{I}}^{\text{RWD}}$	$\pi_{\mathbf{C}}^{\text{RWD}}$	**m**	$\mathbf{d}^{\text{trial}}$	$\hat{\mathbf{\Delta}}^{\text{trial}}$	$\mathbf{B}(\mathbf{d}^{\text{trial}})$	$\mathbf{B}(\hat{\mathbf{\Delta}}^{\text{trial}})$	**gain (%)**
0.7	0.4	112	0.1	0.1	0.4	101	0.30	0.28	-0.00	-0.02	-1
0.7	0.4	112	0.3	0.1	0.4	78	0.30	0.28	-0.00	-0.02	-1
0.7	0.4	112	0.5	0.1	0.4	56	0.30	0.28	-0.00	-0.02	-1
0.7	0.4	112	0.1	0.7	0.4	101	0.30	0.30	-0.00	-0.00	14
0.7	0.4	112	0.3	0.7	0.4	78	0.30	0.30	-0.00	-0.00	13
0.7	0.4	112	0.5	0.7	0.4	56	0.30	0.30	-0.00	-0.00	11
0.7	0.5	248	0.1	0.1	0.5	223	0.20	0.19	-0.00	-0.01	0
0.7	0.5	248	0.3	0.1	0.5	174	0.20	0.19	-0.00	-0.01	0
0.7	0.5	248	0.6	0.1	0.5	99	0.20	0.19	-0.00	-0.01	-1
0.7	0.5	248	0.1	0.7	0.5	223	0.20	0.20	-0.00	-0.00	12
0.7	0.5	248	0.3	0.7	0.5	174	0.20	0.20	-0.00	-0.00	11
0.7	0.5	248	0.6	0.7	0.5	99	0.20	0.20	-0.00	-0.00	8
0.8	0.5	104	0.1	0.2	0.5	94	0.30	0.28	-0.00	-0.02	-1
0.8	0.5	104	0.2	0.2	0.5	83	0.30	0.28	-0.00	-0.02	-1
0.8	0.5	104	0.5	0.2	0.5	52	0.30	0.28	-0.00	-0.02	-1
0.8	0.5	104	0.1	0.8	0.5	94	0.30	0.30	-0.00	-0.00	14
0.8	0.5	104	0.2	0.8	0.5	83	0.30	0.30	-0.00	-0.00	14
0.8	0.5	104	0.5	0.8	0.5	52	0.30	0.30	-0.00	-0.00	11
0.8	0.6	218	0.1	0.2	0.6	196	0.20	0.19	-0.00	-0.01	-1
0.8	0.6	218	0.3	0.2	0.6	153	0.20	0.19	-0.00	-0.01	-1
0.8	0.6	218	0.6	0.2	0.6	87	0.20	0.19	-0.00	-0.01	-1
0.8	0.6	218	0.1	0.8	0.6	196	0.20	0.20	-0.00	-0.00	12
0.8	0.6	218	0.3	0.8	0.6	153	0.20	0.20	-0.00	-0.00	10
0.8	0.6	218	0.6	0.8	0.6	87	0.20	0.20	-0.00	-0.00	8
0.9	0.6	84	0.1	0.3	0.6	76	0.30	0.28	0.00	-0.02	-1
0.9	0.6	84	0.2	0.3	0.6	67	0.30	0.28	0.00	-0.02	-1
0.9	0.6	84	0.5	0.3	0.6	42	0.30	0.28	0.00	-0.02	-1
0.9	0.6	84	0.1	0.9	0.6	76	0.30	0.30	0.00	0.00	14
0.9	0.6	84	0.2	0.9	0.6	67	0.30	0.30	0.00	0.00	13
0.9	0.6	84	0.5	0.9	0.6	42	0.30	0.30	0.00	0.00	11
0.9	0.7	164	0.1	0.3	0.7	148	0.20	0.19	-0.00	-0.01	-1
0.9	0.7	164	0.3	0.3	0.7	115	0.20	0.19	-0.00	-0.01	-1
0.9	0.7	164	0.6	0.3	0.7	66	0.20	0.19	-0.00	-0.01	-1
0.9	0.7	164	0.1	0.9	0.7	148	0.20	0.20	-0.00	-0.00	12
0.9	0.7	164	0.3	0.9	0.7	115	0.20	0.20	-0.00	-0.00	11
0.9	0.7	164	0.6	0.9	0.7	66	0.20	0.20	-0.00	-0.00	8

Table 13: Complement to interim and final clinical trial results for CP when $\pi_I^{\text{trial}} = \pi_C^{\text{trial}}$ over 5,000 replications. Mean and standard deviation of CP value, frequency (on all the 5,000 replications) when the p-value is lower than $\alpha = 0.025$ for the final analysis, frequencies when the CP value is greater than 0.8, 0.85 and 0.9. CP: conditional power; π_I^{trial}, π_C^{trial}: true response rates associated to intervention and control arms for the clinical trial population; n: total number of individuals in the trial; t: information time; pval: p-value for the final analysis.

π_I^{trial}	π_C^{trial}	n	t	CP	pval $\leq \alpha$	CP $\geq$ 0.8 Yes	CP $\geq$ 0.85 Yes	CP $\geq$ 0.9 Yes
0.7	0.7	112	0.5	0.11 (0.23)	0.026	0.040	0.036	0.028
0.7	0.7	112	0.7	0.07 (0.19)	0.026	0.023	0.020	0.013
0.7	0.7	112	0.9	0.04 (0.16)	0.026	0.021	0.017	0.014
0.7	0.7	248	0.5	0.11 (0.23)	0.024	0.037	0.032	0.023
0.7	0.7	248	0.7	0.07 (0.18)	0.024	0.020	0.016	0.013
0.7	0.7	248	0.9	0.04 (0.15)	0.024	0.017	0.014	0.012
0.8	0.8	104	0.5	0.12 (0.23)	0.024	0.040	0.030	0.028
0.8	0.8	104	0.7	0.07 (0.19)	0.024	0.024	0.020	0.016
0.8	0.8	104	0.9	0.04 (0.16)	0.024	0.018	0.015	0.014
0.8	0.8	218	0.5	0.11 (0.22)	0.026	0.031	0.025	0.020
0.8	0.8	218	0.7	0.07 (0.18)	0.026	0.021	0.017	0.013
0.8	0.8	218	0.9	0.04 (0.15)	0.026	0.018	0.015	0.012
0.9	0.9	84	0.5	0.12 (0.24)	0.026	0.057	0.023	0.022
0.9	0.9	84	0.7	0.08 (0.19)	0.026	0.024	0.018	0.009
0.9	0.9	84	0.9	0.04 (0.16)	0.026	0.018	0.012	0.012
0.9	0.9	164	0.5	0.11 (0.23)	0.026	0.031	0.031	0.019
0.9	0.9	164	0.7	0.07 (0.18)	0.026	0.021	0.014	0.013
0.9	0.9	164	0.9	0.04 (0.16)	0.026	0.019	0.016	0.014

5 AVA-PREPA case study

To illustrate our approach, we use the AVA-PREPA trial as a case study. AVA-PREPA is a randomized controlled trial designed to assess the impact of the AXOMOVE THERAPY (a medical telemonitoring software) on patients with subacute or chronic low back pain discharged from rehabilitation centers, in order to facilitate self-rehabilitation. The primary endpoint of the study was the patient's functional capacity measured by the Oswestry Disability Index (ODI) version 2. 1a. at 105 days. A difference of five points was expected between the two treatment arms. It was calculated that a sample size of 228 patients was needed to detect such a difference in mean score, with a standard deviation of 11, a two-sided test at 5%, a power of 90% and anticipating a lost to follow-up rate of 10%. The chosen allocation ratio (intervention arm to control arm) was 1:1. At the time of submission of the dossier to PECAN, on September 26th 2024, inclusions were still in progress, with 190 patients included (https://www.has-sante.fr/jcms/p_3552523/fr/axomove-therapy).

To be consistent with our main manuscript, we considered $\alpha = 0.025$ for the following one-sided test: $H_0 : \mu_I - \mu_C = 0$ versus $H_A : \mu_I - \mu_C < 0$, and we use $c^* = 0.8$ as the threshold for the conditional power.

In our work, the intervention and control responses $\boldsymbol{x}_I^{\text{trial}}$ and $\boldsymbol{x}_C^{\text{trial}}$ were simulated using normal distributions with respective means $\mu_I^{\text{trial}} = 25$ and $\mu_C^{\text{trial}} = 30$, and respective standard deviations $\sigma_I^{\text{trial}} = 11$ and $\sigma_C^{\text{trial}} = 11$.

At the interim time $t = n_t/n = 190/228 \approx 0.83$, the observed means in the two arms were equal to $\bar{x}_{I,t}^{\text{trial}} \approx 26$ (sd: 9.9) and $\bar{x}_{C,t}^{\text{trial}} \approx 30$ (sd: 11). Then, the conditional power CP was approximately equal to 0.98. Since $CP \geq c^*$, we can apply for regulatory temporary authorization.

At the end of the clinical trial, the null hypothesis that the means in the two arms would be the same was tested with a confidence level equal to $1 - \alpha$. The p-value associated to the test was approximately equal to 0.003. Since it was less than 0.025, the test would be significant and the null hypothesis would be rejected.
